# Diagnostics and treatments of COVID-19: two-year update to a living systematic review of economic evaluations

**DOI:** 10.3389/fphar.2023.1291164

**Published:** 2023-11-16

**Authors:** Jamie Elvidge, Gareth Hopkin, Nithin Narayanan, David Nicholls, Dalia Dawoud

**Affiliations:** ^1^ Science, Evidence and Analytics Directorate, National Institute for Health and Care Excellence, Manchester, United Kingdom; ^2^ Norwich Medical School, University of East Anglia, Norwich, United Kingdom

**Keywords:** cost-effectiveness, COVID-19, diagnostics, economic evaluation, health technology assessment, pharmacological, living review, cost-utility analysis

## Abstract

**Objectives:** As the initial crisis of the COVID-19 pandemic recedes, healthcare decision makers are likely to want to make rational evidence-guided choices between the many interventions now available. We sought to update a systematic review to provide an up-to-date summary of the cost-effectiveness evidence regarding tests for SARS-CoV-2 and treatments for COVID-19.

**Methods:** Key databases, including MEDLINE, EconLit and Embase, were searched on 3 July 2023, 2 years on from the first iteration of this review in July 2021. We also examined health technology assessment (HTA) reports and the citations of included studies and reviews. Peer-reviewed studies reporting full health economic evaluations of tests or treatments in English were included. Studies were quality assessed using an established checklist, and those with very serious limitations were excluded. Data from included studies were extracted into predefined tables.

**Results:** The database search identified 8,287 unique records, of which 54 full texts were reviewed, 28 proceeded for quality assessment, and 15 were included. Three further studies were included through HTA sources and citation checking. Of the 18 studies ultimately included, 17 evaluated treatments including corticosteroids, antivirals and immunotherapies. In most studies, the comparator was standard care. Two studies in lower-income settings evaluated the cost effectiveness of rapid antigen tests and critical care provision. There were 17 modelling analyses and 1 trial-based evaluation.

**Conclusion:** A large number of economic evaluations of interventions for COVID-19 have been published since July 2021. Their findings can help decision makers to prioritise between competing interventions, such as the repurposed antivirals and immunotherapies now available to treat COVID-19. However, some evidence gaps remain present, including head-to-head analyses, disease-specific utility values, and consideration of different disease variants.

**Systematic Review Registration:** [https://www.crd.york.ac.uk/prospero/display_record.php?ID=CRD42021272219], identifier [PROSPERO 2021 CRD42021272219].

## 1 Introduction

The novel severe acute respiratory syndrome coronavirus 2 (SARS-CoV-2) and its associated disease (COVID-19) pandemic placed healthcare systems and wider economies under massive strain in 2020 and 2021. Decisions about diagnostic tests and treatments for the disease were made rapidly, forgoing traditional, rigorous health technology assessments (HTAs) that healthcare interventions are subjected to in many countries. Now that the early pandemic crisis has passed, HTA organisations will increasingly view COVID-19 as being equivalent to any other condition, and seek to understand the *cost* effectiveness of tests and treatments for it. Such evidence can support reimbursement decisions and the efficient allocation of scarce healthcare resources.

In July 2021, the first iteration of a systematic literature review to identify economic evaluations of tests and treatments for COVID-19 was conducted (Elvidge et al., 2022). Its objective was to identify up-to-date cost-effectiveness estimates for COVID-19 tests and treatments, and the methodological approaches, limitations and uncertainties present in published economic evaluations. Since then, the pandemic context, evidence base, and disease have evolved considerably. The present study reports a timely two-year update of the review, to provide a contemporary understanding of the cost-effectiveness evidence for COVID-19 tests and treatments.

This study has been supported by Next-Generation Health Technology Assessment (HTx), which is a Horizon 2020 project supported by the European Union, lasting for 5 years from January 2019. Its main aim is to create a framework for the next-generation of HTA to support patient-centred, societally oriented, real-time decision making on access to and reimbursement for health technologies throughout Europe.

## 2 Materials and methods

We performed an update of a previously published systematic literature review to identify full economic evaluations of diagnostics (e.g., tests) for SARS-CoV-2 and treatments (e.g., pharmaceuticals) for COVID-19 (Elvidge et al., 2021; Elvidge et al., 2022). The date range spanned the previous search date, 12th July 2021, to 3rd July 2023. The search strategy was consistent with the original search, including citation checking of included studies and efforts to identify relevant grey literature. Studies were included if they were full economic evaluations, comparing both the costs and health outcomes of 2 or more alternative tests for SARS-CoV-2 or treatments for COVID-19.

Every identified title and abstract was screened against the selection criteria by 2 reviewers (JE and NN/GH). For studies that were identified as potentially relevant, full-text articles were sought and assessed against the selection criteria by both reviewers. Studies that met the selection criteria were quality assessed by both reviewers, using the NICE economic evaluation quality checklist (National Institute for Health and Care Excellence NICE, 2012). Those judged to have very serious limitations were excluded. For each included study, data extraction was conducted by 1 reviewer using prespecified tables consistent with the original review. Extracted data for each study were checked and validated by another reviewer. At all stages, discrepancies between the reviewers were resolved through discussion or, if needed, adjudication by a senior reviewer (DD). Key study characteristics are presented in Tables 1, 2, and findings in Table 3. Due to extensive heterogeneity between studies, results were synthesised narratively (Shields and Elvidge, 2020).

**TABLE 1:** General characteristics of included studies.

Study	Country (currency)	Population/Setting	Intervention(s) & comparator(s)	Type of evaluation	Quality assessment^b^
Alamer 2023 (Alamer et al., 2023)	Saudi Arabia (SAR)	415 patients with moderate to severe COVID-19 disease who were admitted to two Saudi COVID-19 referral hospitals	Favipiravir, standard of care (SoC)	CEA	Potentially serious limitations
Arwah 2023 (Arwah et al., 2023)	Kenya (USD)	Patients with suspected COVID-19 presenting at settings with access to point-of-care testing	Two comparisons	CUA	Potentially serious limitations
			Rapid tests with delayed confirmatory testing for negative, delayed testing		
			Rapid tests, clinical judgement		
Carta and Conversano 2021 (Carta and Conversano, 2021)	United States (USD)	Hospitalised COVID-19 patients (4 levels of respiratory support), aged 60	Remdesivir, dexamethasone, remdesivir + dexamethasone (R + D), SoC	CUA	Potentially serious limitations
Congly 2021 (Congly et al., 2021)	United States (USD)	Hospitalised patients, moderate (oxygen) & severe (ICU), aged 60	Combinations of SoC, redemsivir and dexamethasone, by severity	CUA	Potentially serious limitations
Dijk 2022 (Dijk et al., 2022)	United States (USD)	Hospitalised COVID-19 patients	Hydroxychloroquine, remdesivir, casirivimab + imdevimab (C + I), dexamethasone, baricitinib + remdesivir (B + R), tocilizumab, lopinavir + ritonavir (L + R), interferon b1a, SoC	CUA	Minor limitations
Goswami 2022 (Goswami et al., 2022)	United States (USD)	Outpatient adults with mild to moderate COVID-19 and 1 or more risk factor for severe disease	Molnupiravir, SoC	CUA	Minor limitations
Kelton 2021 (Kelton et al., 2022)	United States (USD)	Hospitalised COVID-19 patients	B + R, remdesivir	CUA	Potentially serious limitations
Kowal 2023 (Kowal et al., 2023)	United States (USD)	Hospitalised COVID-19 patients, stratified into equity-relevant subgroups by race/ethnicity and deprivation	Hypothetical treatment, SoC (per clinical trials in 2020)	DCUA	Potentially serious limitations
Lau 2022 (Lau et al., 2022)	Canada (CAD)	Adult, hospitalized patients with COVID-19	Remdesivir, SoC	CEA	Minor limitations
Metry 2022 (Metry et al., 2022)	United Kingdom (GBP)	In hospital or in community and at high risk of hospitalisation	Hospital setting	CUA	Minor limitations
			Baricitinib, B + R, C + I^a^, lenzilumab^a^, remdesivir, tocilizumab, SoC		
			Community setting		
			C + I^a^, molnupiravir^a^, nirmatrelvir + ritonavir (N + R), remdesivir, sotrovimab, SoC		
Park 2022 (Park et al., 2022)	Singapore (USD)	4 relevant scenarios of unvaccinated patients by age group	C + I, SoC	CEA	Potentially serious limitations
				CUA	
Rafia 2022 (Rafia et al., 2022)	United Kingdom (GBP)	Hospitalised COVID-19 patients requiring oxygen or non-invasive ventilation (NIV)	Remdesvir, SoC	CUA	Minor limtations
Ruggeri 2022 (Ruggeri et al., 2022a)	Portugal (EUR)	Hospitalised COVID-19 patients on low-flow oxygen	Remdesivir, SoC	CEA	Potentially serious limitations
Ruggeri 2022 (Ruggeri et al., 2022b)	Saudi Arabia (USD)	Hospitalised COVID-19 patients on low-flow oxygen	Remdesivir, SoC	CEA	Potentially serious limitations
Ruggeri 2023 (Ruggeri et al., 2023)	Italy (EUR)	Outpatients with COVID-19 not having low-flow oxygen	C + I, SoC	CEA	Potentially serious limitations
Savinkina 2022 (Savinkina et al., 2022)	United States (USD)	Newly diagnosed COVID-19 positive patients, including subgroups by high & low risk of severe disease and vaccination status (vaccine assumed to be 75% effective at reducing hospital risk)	N + R; SoC (no N + R); and 3 interim stratgies with different levels of N + R	CEA	Potentially serious limitations
Shah 2023 (Shah et al., 2023)	Tanzania (USD)	Hospitalised critically ill adult patients with COVID-19	Advanced critical care, essential critical care, district-level critical care, no critical care	CUA	Potentially serious limitations
Yeung 2022 (Yeung et al., 2022)	United States (USD)	Mild to moderate outpatients at high risk of progression to severe disease	Molnupiravir, N + R, fluvoxamine, SoC (pooled from key trials)	CUA	Minor limitations

^a^ In Metry et al., the cost-effectiveness results for lenzilumab, molnupiravir and C + I were considered to be confidential and were therefore not made publicly available.

^b^ Minor limitations indicates the study meets all quality assessment criteria, or fails 1 or more criteria but this is unlikely to change the conclusions about cost effectiveness. Potentially serious limitations indicates the study fails 1 or more quality assessment criteria and this has the potential to change the conclusions about cost effectiveness.

Abbreviations: B + R, baricitinib + remdesivir; CEA, cost-effectiveness analysis; CUA, cost—utility analysis; C + I, casirivimab + imdevimab; DCUA, distributional cost—utility analysis; ICU, intensive care unit; L + R, lopinavir + ritonavir; N + R, nirmatrelvir + ritonavir; NIV, non-invasive ventilation; SoC, standard of care.

**TABLE 2:** Economic evaluation characteristics of included studies.

Study	Analysis approach	Perspective	Time horizon	Cost categories	Cost year	Discounting	Health outcomes	Efficacy data source	Utility data source
Alamer 2023 (Alamer et al., 2023)	Patient-level simulation	Healthcare payer	5 months	Favipiravir, inpatient care, isolation room, personnel, laboratory, tests	2020	NR	Deaths averted	Retrospective comparative study using propensity score matching	NA
Arwah 2023 (Arwah et al., 2023)	Decision tree	Societal	Patient care episode	Testing, treatment, related healthcare services, isolation, travel, value of time, informal care, productivity loss	2021	3%	DALYs averted	Author assumptions and observational evidence	Non-COVID sources
Carta and Conversano 2021 (Carta and Conversano, 2021)	Decision tree	Healthcare	1 year	Treatments, inpatient care (by LoS), follow-up care	NR (appears to be 2020)	NA	QALY	RCTs. Remdesivir and dexamethasone effects assumed to be additive	Non-COVID sources
Congly 2021 (Congly et al., 2021)	Decision tree	Healthcare	1 year	Treatments, inpatient care (by DRG)	2020	NA	QALY	Meta analysis & RCT	Non-COVID sources
Dijk 2022 (Dijk et al., 2022)	Markov model	Healthcare	Lifetime	Treatments, inpatient care, rehabilitation	2020	3%	QALY	RCTs	Non-COVID sources
Goswami 2022 (Goswami et al., 2022)	Hybrid model: decision tree followed by Markov	Healthcare	Lifetime	Molnupiravir, inpatient care, outpatient care, emergency care	2021	3%	QALY	RCT	Primary vignettes study, EQ-5D-5L (United Kingdom n = 500) using US value set.
Kelton 2021 (Kelton et al., 2022)	Hybrid model: decision tree followed by Markov	Base case: partial societal (hospital plus indirect productivity costs). Scenario: hospital only	Base case: lifetime. Hospital scenario: hospitalisation duration	Treatments, inpatient care (base case: by LoS; hospital scenario: less DRG payments); post-discharge care; long-term all-cause care; lost work days	NR	3%	QALY	RCTs. “Data on file” cited for the ACTT-2 trial	Non-COVID sources
Kowal 2023 (Kowal et al., 2023)	Distributional reanalysis of Sheinson 2021 model	Healthcare (payer)	Same as Sheinson 2021						
Lau 2022 (Lau et al., 2022)	Trial-based	Healthcare public payer	To discharge or death	Remdesivir, ICU & ward stays, personnel, laboratory and radiology, procedures, surgeries	2020	None	Deaths averted	RCT	NA
Metry 2022 (Metry et al., 2022)	Hospital: partitioned survival model Community: decision tree model	Healthcare	Lifetime	Treatments, hospital care, outpatient monitoring, long COVID	NR	NR	QALY	Living NMAs (metaEvidence Initiative, 2022; The COVID-NMA Initiative, 2021)	Non-COVID sources
Park 2022 (Park et al., 2022)	Decision tree	NR	Duration of illness	C + I, hospital care	NR	NA	Deaths averted, DALYs averted	RCTs	Burden on illness study in Malta, derived from non-COVID disability weights
Rafia 2022 (Rafia et al., 2022)	Partitioned survival model	Healthcare	Lifetime	Treatments, hospital care	NR	3.5%	QALY	RCT	Non-COVID sources
Ruggeri 2022 (Ruggeri et al., 2022a)	Markov model nested within epidemiological model	Hospital	20 weeks	Remdesivir, inpatient costs including ICU	NR	NA	Deaths averted	RCT	NA
Ruggeri 2022 (Ruggeri et al., 2022b)	Markov model nested within epidemiological model with 3 scenarios: Static, decreasing and increasing infection rates	NR	20 weeks	Remdesivir, inpatient costs including ICU and IV	2020	NA	Deaths averted	RCT	NA
Ruggeri 2023 (Ruggeri et al., 2023)	Markov model nested within epidemiological model	Healthcare (payer)	20 weeks	C + I, inpatient costs including ICU	NR	NA	Deaths averted	RCT	NA
Savinkina 2022 (Savinkina et al., 2022)	Decision tree model	Healthcare	30 days	N + R, admission cost	NR	NA	Deaths averted	High risk, not vaccinated: RCT and observational data. Not high risk or high risk and vaccinated: RCT.	NA
Shah 2023 (Shah et al., 2023)	Markov model	Provider perspective	28 days	Cost of critical care (limited details)	2020	None	DALYs averted	Expert elicitation	Non-COVID sources
Yeung 2022 (Yeung et al., 2022)	Hybrid model: decision tree followed by Markov	Base case: healthcare Scenario:modified societal	Lifetime	Treatments, related care, age-adjusted other healthcare, productivity costs (scenario)	2021	3%	QALY	RCTs. Manufacturer press release cited for N + R trial	Non-COVID sources

Abbreviations: DALY, disability-adjusted life-year; DRG, diagnostic-related group; EQ-5D-5L, Euroqol 5 dimension 5 level; ICU, intensive care unit; LoS, length of stay; MV, mechanical (invasive) ventilation; NA, not applicable; NMA, network meta-analysis; NR, not reported; QALY, quality-adjusted life-year; RCT, randomised controlled trial.

**TABLE 3:** Results of included studies.

Study	Cost and health outcome results	ICER/net benefit of intervention(s) vs. comparator(s)	Cost-effectiveness threshold (if relevant)	Sensitivity & scenario analyses	Authors’ conclusions regarding cost effectiveness	Authors’ reported limitations and challenges
Alamer 2023 (Alamer et al., 2023)	Favipiravir: $17,197*; 0.97 probability survival	-$4,534* per death averted	No threshold reported	Analysis replicated with weighted model using propensity scores and PSA completed for weight and unweighted analyses	Favipiravir was associated with lower cost than SoC in both the unweighted and the weighted models. Favipiravir was also associated with a higher probability to be discharged alive	Limiting to deaths averted and time to discharge may miss important outcomes. Extending to CUA was not possible but would be desirable
	SoC: $35,331*; 0.93 probability survival		Report no agreed thresholds in Saudi but dominant	Favipiravir is less costly and more effective across all analyses		Study did not explore timing of treatment which may be important
Arwah 2023 (Arwah et al., 2023)	Scenario 1 (access to confirmatory testing)	Scenario 1	$1003 (stated as Kenyan threshold)	Deterministic sensitivity with key parameters varied and PSA	Using rapid testing as a first-line tool, and later confirmatory tests of negatives where available, was a cost-effective strategy. Otherwise, rapid testing is preferred to clinical judgement, although it is less costly and less effective	Limited by unavailable data on outcomes for false negative patients
	Rapid testing with delayed confirmatory testing: $1,336,231, 1999 DALYs	$965 per DALY averted (rapid testing more costly and more effective)		Cost-effectiveness was sensitive to changes in the prevalence, changes to sensitivity and specificity of rapid testing and confirmatory testing		
	Delayed testing: $1,107,118, 2236 DALYs	Scenario 2		Scenario 1:		
	Scenario 2 (no access to confirmatory testing)	$1490 per DALY averted (clinical judgement more costly and more effective)		Rapid testing had probability of 52.5% of being cost-effective at threshold		
	Rapid testing: $998,260.67, 2538 DALYs			Scenario 2:		
	Clinical judgement: $1,261,230, 2361 DALYs			Rapid testing had probability of 71% of being cost-effective at threshold		
Carter & Conversano 2021 (Carta and Conversano, 2021)	SoC: $33,370, 0.767 QALYs	R + D dominates both SoC and dexamethasone	$50K/QALY gained	OWSA results presented vs. SoC only. All ICERs vs. SoC robust except when remdesivir relative effect takes lower bound estimate (R + D: $24.4K/QALY, remdesivir: $261K/QALY)	This analysis supports the use of remdesivir and/or dexamethasone	Analysis is based on limited evidence of treatment effectiveness
	Remdesivir: $32,354, 0.773 QALYs	R + D vs. remdesivir: $5,222/QALY		PSA results consistent with base case		R + D does not have proven effectiveness
	Dexamethasone: $33,556, 0.803 QALYs	Excluding R + D:				Disease progression, long-COVID and different patient characteristics not explored
	R+D: $32,540, 0.809 QALYs	Remdesivir dominates SoC				Proxy utility data used
		Dexamethasone vs remdesivir: $40.6K/QALY				
Congly 2021 (Congly et al., 2021)	Total costs & QALYs	All ICERs for remdesivir strategies are dominated by giving dexamethasone to all (moderate and severe) patients	$100K/QALY gained	Optimal strategy is not sensitive to OWSA	Dexamethasone for all patients was the most cost-effective strategy Dexamethasone for severe cases would be favoured at lower decision thresholds	Analysis is based on limited evidence of treatment effectiveness
	(Strategies denoted by treatment for moderate disease, treatment for severe disease.)	Dexamethasone for severe only, vs. SoC: $285/QALY		PSA: ICER for dexamethasone (all patients) is below £100K/QALY with 98% probability		Fixed DRG costs do not account for different hospital stay durations
	1. SoC, SoC: $11.1K, 0.716	Dexamethasone for all patients vs. severe only: $1,718/QALY				No long-term health outcomes
	2. SoC, Dex: $11.1K, 0.726					Proxy utility data used
	3. Dex, Dex: $11.1K, 0.735					
	4. SoC, Rem: $11.8K, 0.710					
	5. Rem, SoC: $13.1K, 0.725					
	6. Rem, Dex: $13.1K, 0.734					
	7. Rem, Rem: $13.7K, 0.719					
Dijk 2022 (Dijk et al., 2022)	Incremental vs. SoC	ICERs vs. SoC	$100K/QALY gained	Value of information analysis	At a threshold of $100K/QALY gained, treatment with remdesivir, C + I, dexamethasone, B + R and tocilizumab are cost effective versus SoC	Some parameters were estimated from non-COVID studies. Effectiveness estimates drawn from single trials
	Hydroxychloroquine (Hyd): -$12,227, -0.263 QALYs	Hyd: $46,427 (SWQ)		Decisions about Dex, C + I, B + R, L + R and IF would not change with		Analysis focuses on the research and approval health policy questions, not comparisons
	Remdesivir (Rem): -$5, +0.252 QALYs	Rem: Dominant		further evidence		
	C + I: $696, +0.171 QALYs	C + I: $4,075		For Rem and Toc, the value of further evidence would not outweigh the cost of research		
	Dexamethasone (Dex): $6856, +0.614 QALYs	Dex: $11,619		For Hyd, further evidence to investigate decremental cost effectiveness may be worthwhile		
	B + R: $10,673, +0.775 QALYs	B + R: $13,772				
	Tocilizumab (Toc): $35,849, +0.882 QALYs	Toc: $40,633				
	Interferon b1 (IF): -$2,538, -0.472 QALYs	IF: $5,377 (SWQ)				
	L + R: -$1,404, -0.091 QALYs	L + R: $15,418 (SWQ)				
		Fully incremental NR due to heterogeneous SoC arms across trials				
Goswami 2022 (Goswami et al., 2022)	Molnupiravir: $8,795, 17.721 QALYs	Molnupiravir is dominant compared with SoC	$100K per QALY gained	Results robust to scenario and one-way sensitivity analyses. PSA: Molnupiravir 100% likely to have an ICER below threshold	Compared with SoC, treatment with molnupiravir can be considered a cost-effective option in the management of outpatients with COVID-19 at risk of progression to severe disease in the US	Other outpatient treatments for COVID-19 not included. Appropriate utility data were unavailable and required primary research
	SoC: $9,690, 17.512 QALYs					
Kelton 2022 (Kelton et al., 2022)	Partial societal perspective	Partial societal perspective	$50K/QALY gained	All ICERs robust to OWSA, including oxygen/NIV subgroup	B + R is more cost effective than remdesivir alone for patients hospitalised because of COVID-19 in the US	Lack of data to inform long-term burden of COVID-19
	Remdesivir: $372K, 11.7 QALYs	B + R vs. remdesivir: $22.3K/QALY, $17.9K/LYG		B + R more cost effective if no survival benefit (due to future unrelated medical costs avoided)		Analysis does not capture potential readmissions or resource capacity constraints
	B + R: $380K, 12.1 QALYs	Hospital perspective		PSA: consistent with deterministic		Data informing utility values are limited
	Note: >80% of costs composed of other long-term medical costs	B + R dominates remdesivir				National average DRG costs may lack generalisability
	Hospital perspective					
	B + R vs. remdesivir: -$1,778, +0.0018 QALYs					
Kowal 2023 (Kowal et al., 2023)	Deterministic incremental results	Deterministic results	$150,000 per QALY gained ($50K & $100K in sensitivity analyses)	Population NHB by threshold:	Funding COVID-19 treatments reduced the population-level burden of health inequality by 0.234% (or 130,000 QALYs)	Underreporting of COVID-19 cases, hospitalisations deaths, and potentially variable reporting across equity subgroups. 20% of the population was not captured by the DCUA
	Average:	Average: $28,600 per QALY gained		$50K: 391,114 QALYs	Distributional CUA of inpatient COVID-19 treatments may improve overall health while reducing health inqualities	No trial data were identified that reported subgroup effects
	Costs $12,741, QALYs +0.445	Highest deprivation: $28,000 per QALY gained		$100K: 649,456 QALYs		Lack of quality-of-life data at the subgroup level
		Lowest deprivation: $29,800/QALY gained		Population NHB remain positive up to inpatient treatment cost of $60,100 per patient		
		Including inequitable opportunity costs (NHB)				
		Total: 735,569 QALYs				
		Hispanic, highest deprivation: 72,083; lowest deprivation: 1,106				
		Black, highest deprivation: 47,342; lowest deprivation: 1,622				
		White, highest deprivation: 113,982; lowest deprivation: 27,450				
Lau 2022 (Lau et al., 2022)	Remdesivir: $28,276*, 0.809 deaths averted	Dominant	Thresholds of $0, $14,914*, $37,286* and $74,571* used for interpreting PSA	Results similar across deterministic scenarios. Major drivers of cost effectiveness were inpatient care and remdesivir costs	Remdesivir plus SoC is likely the preferred treatment strategy compared with usual care alone, for hospitalised adults with COVID-19	Short time horizon may miss downstream costs and later events
	Placebo: $28,357*, 0.771 deaths averted			Remdesivir dominant in 58% of PSA simulations and below $74,571* in 82%		Data from RCT may not reflect routine clinical practice
Metry 2022 (Metry et al., 2022)	Total costs* & QALYs	In hospital, on oxygen:	$27.5K* /QALY gained	Treatments are more cost effective when duration of long COVID was shorter, and in younger patients. In the community setting, a higher risk of hospitalisation makes early treatment more cost effective	In hospital, all treatments evaluated had scenarios where the ICER vs. SoC was below the threshold	The decision problem has evolved, so studies do not reflect the current conditions. Therefore, many assumptions were required. No head-to-head studies of interventions were identified. Confidential results not published for lenzilumab, molnupiravir or casirivimab + imdevimab
	In hospital, on oxygen:	SoC: reference			In the community setting, N + R may be cost effective compared with SoC	
	SoC: $30,436, 4.61	T: $9,254*				
	Toc: $35,146, 5.12	Rem: dominated				
	Rem: $38,202, 5.08	Bar: $18,812*				
	Baricitinib (Bar): $41,572, 5.46	B + R: dominated				
	B + R: $41,974, 5.32	In hospital, no oxygen:				
	In hospital, no oxygen:	SoC: reference				
	SoC: $13,316, 5.79	Bar: $7,564*				
	Bar: $16,073, 6.29	Rem: dominated				
	Rem: $16,487, 6.07	B + R: dominated				
	B+R: $17,509, 6.21	In the community, high risk:				
	In the community, high risk:	SoC: reference				
	SoC: $1,448, 13.42	N + R: $8,484*				
	N+R: $2,483, 13.53	Sot: dominated				
	Sot: $4,924, 13.48	Rem: dominated				
	Rem: $6,039, 13.45					
Park 2022 (Park et al., 2022)	Incremental results (costs and DALYs averted)	Treatment with C + I vs. SoC:	1.15 gross national income per DALY = $74K in 2021	Results were robust to sensitivity analyses setting the relative risk reduction to the 95% CI bounds	All strategies considered were cost effective using the specified threshold	Study prior to widespread circulation of delta and omicron disease variants. Efficacy and cost effectiveness of C + I may differ by variant
	Treatment with C+I vs SoC:	Dominant			Treating people aged ≥60 was the most cost saving strategy	
	Treat ages ≥80: -$0.08 m, 38	Dominant				
	Treat ages ≥70: -$0.1 m, 66	Dominant				
	Treat ages ≥60: -$0.34 m, 161	$800/DALY averted				
	Treat ages ≥50: +$0.17 m, 198					
Rafia 2022 (Rafia et al., 2022)	Total costs* and QALYs (probabilistic)	If remdesivir has a survival effect:	$27.5K* /QALY gained	ICERs most affected by time horizon, baseline survival with SoC, and inclusion of unrelated costs. At analysis price, remdesivir mortality HR must be 0.915 or higher to be cost effective	Remdesivir is likely to be cost effective only if it prevents death, and this is highly uncertain within the supplemental oxygen population	Rapidly changing context means some parameter estimates and assumptions out of date
	If remdesivir has a survival effect:	ICER vs. SoC: $17,056*		PSA: ICER below threshold with 74% probability if it confers a survival benefit, else 0%		Model cannot track individual patients
	SoC: $12,920, 6.35	If remdesivir has no survival effect:				Analyses conducted at list prices, may not reflect true prices paid
	Remdesivir: $17,549, 6.62	ICER vs. SoC: >$1M.				Potentially some double counting of COVID-19 disutility
	If remdesivir has no survival effect:					
	SoC: $14,190, 6.35					
	Remdesivir: $16,481, 6.35					
Ruggeri 2022 (Ruggeri et al., 2022a)	Incremental results	NR	NR	Results sensitive to Rt; admission, ICU and mortality rates; remdesivir treatment effect. However, conclusions remain the same	The ability of remdesivir to decrease ward LoS and ICU admissions would produce signifcant cost savings for hospitals, a more manageable hospital capacity in a public health emergency, and a faster recovery for hospitalised patients who require supplemental oxygen	Infection forecasts were informed by various sources, including historical data and expert opinion, and are therefore uncertain. Potential side effects of remdesivir were not included
	23,579 cases:			PSA: results not reported in detail, but remdesivir appears to be cost-incurring (i.e., not dominant) in a significant proportion of PSA results		
	Costs -$27.8 m*					
	Deaths averted 165.9					
	Calibrated to 1,000 cases:					
	Costs -$1.2 m*					
	Deaths averted 7.0					
Ruggeri 2022 (Ruggeri et al., 2022b)	Incremental results	NR	NR	Results sensitive to Rt values, ICU and mortality rates, baseline hospitalisation and remdesivir mortality effect, but conclusions remain the same	In Saudi Arabia, remdesivir plus standard of care has the potential to reduce healthcare resource use, mortality, and costs when compared with	Some infection forecasts were informed by expert opinion, and are therefore uncertain. Many inputs informed by targeted, rather than systematic, literature review, including only 1 RCT. Treatment-related adverse eventsnot captured
	Static infection rate (178,405 cases):			Rt = 0.8 (decreasing; 109,087 cases): costs -$154.7 m^1^, DA 815	standard of care alone across a range of plausible local epidemiological scenarios	
	Costs -$174.81 m			Calibrated to 1,000 cases: costs -$1.4 m, DA 7.5		
	Deaths averted (DA) 1.2			Rt = 1.2 (increasing; 247,724 cases): costs -$377.3 m, DA 1,582		
	Calibrated to 1,000 cases:			Calibrated to 1,000 cases: costs -$1.5 m, DA 6.4		
	Costs -$979,836			PSA: remdesivir is dominant in 93% of simulations		
	Deaths averted 6.7					
Ruggeri 2023 (Ruggeri et al., 2023)	Incremental results	NR	NR	Results sensitive to Rt; admission, ICU and mortality rates; C + I effect on admissions. However, conclusions remain the same	[With C + I] hospitals can achieve important cost savings while patients can experience a more favourable disease course [including reduction in death]	Epidemiological model based on estimated parameters, including Rt. Limited clinical evidence about C + I (1 RCT). True price of C + I in Italy is not known, therefore this analysis uses the US price. Dominant COVID-19 variants at the time of publication (alpha and delta) are not the variant that C + I is likely to be active against (omicron; prevalence 4.76%)
	194,451 cases:			PSA: C + I dominant in more than 90% of simulations		
	Costs -$82.4 m*					
	Deaths averted 1,535					
	Calibrated to 1,000 cases:					
	Costs -$423,730*					
	Deaths averted 7.9					
Savinkina 2022 (Savinkina et al., 2022)	Base-case (high) effect scenario, calibrated to 1,000 patients:	Base-case (high) effect scenario:	$10,000 to $5 m per DA	ICERs, low-effect scenario:	For almost every scenario prescribing N + R to unvaccinated patients at high risk of severe COVID-19 was cost saving. This group should almost always be treated if treatment is available	Analysis does not consider drug supply, budgetary constraints, non-adherence, contraindications to N + R, other active treatments, differential costs in different vacc and risk groups, or transmission dynamics
	No N + R: $221K, 0.77 deaths	No N + R: baseline		No N + R: baseline		
	N + R for unvacc high risk: $182K, 0.51 deaths	N + R for unvacc high risk: dominant		N + R for unvacc high risk: $319K per DA		
	N + R for all high risk: 0.29 deaths, $273K	N + R for all high risk: $397K per DA		N + R for all high risk: $2.6 m per DA		
	N + R for all high risk and unvacc low risk: 0.22 deaths, $348K	N + R for all high risk and unvacc low risk: $1.0 m per DA		N + R for all high risk and unvacc low risk: $5.3 m per DA		
	N + R for all: 0.18 deaths, $566K	N + R for all: $5.0m per DA		N + R for all: $22.1m per DA		
				Cost results reported for various OWSA values (but ICERs NR)		
Shah 2023 (Shah et al., 2023)	Not reported (incremental only)	Advanced CC vs none:	$101 per DALY averted (conservative threshold for Tanzania)	Probability of essential and emergency care being cost effective is 96% and 99% compared to no care and district level care at Tanzanian threshold	Essential and emergency critical care is likely to be highly cost effective in low-resource settings	Analysis relies on low quality sources for parameters due to scarcity of data, does not include needs of moderate patients, and did not reflect availability of regional and referral hospitals
		$186 per DALY averted		In deterministic analyses, results were most sensitive to effectiveness of essential and emergency care in preventing severe cases becoming critical, unit costs of advanced care		Markov model cannot capture pace of change of treatment even within 24 h cycle
		Essential CC vs none:				Triangular distributions used may be less appropriate but reflect uncertain nature of data
		$37 per DALY averted				
		Advanced CC vs district:				
		$144 per DALY averted				
		Essential CC vs. district:				
		$14 per DALY averted				
Yeung 2022 (Yeung et al., 2022)	Costs and QALYs	ICERs vs. SoC	$50K-150K per QALY gained	PSA (healthcare perspective), probability ICER < $50K, $100K, $150K:	At their current prices, each intervention is estimated to meet standard cost-effectiveness levels in the US healthcare system, even under a scenario with a lower hospitalisation risk that may reflect the Omicron wave	Analysis underpinned by immature evidence base and heterogenous trial designs, including non-US settings and different prevalent COVID-19 variants
	Healthcare perspective	Healthcare perspective		Mol: 31%, 69%, 84%		Modified societal perspective has limited scope
	Molnupiravir (Mol): $298.5K, 15.938	Mol: $61K		N + R: 97%, 100%, 100%		
	N + R: $298.5K, 15.964	N + R: $21K		Flu: 100%, 100%, 100%		
	Fluvoxamine (Flu): $297.8K, 15.939	Flu: $8K		Key scenarios, (ICERs vs. SoC):		
	SoC: $297.7K, 15.925	Modified societal perspective (approx.)		Unvaccinated population:		
	Modified societal perspective	Mol: $43K		Mol: $48K		
	Mol: $301.4K, 15.952	N + R: $26K		N + R: $15K		
	N + R: $302.3K, 16.006	Flu: $20K		Flu: $4K		
	Flu: $300.8K, 15.954			Lower hospitalisation risk (e.g., Omicron variant):		
	SoC: $300.2K, 15.925			Mol: $74K		
				N + R: $34K		
				Flu: $21K		

Abbreviations: Bar, baricitinib; B + R, baricitinib and remdesivir; CC, critical care; CUA, cost—utility analysis; C + I, casirivimab + imdevimab; DALY, disability-adjusted life-year; DA, DALY averted; DCUA, distributional cost—utility analysis; Dex, dexamethasone; DRG, diagnostic-related group; Flu, fluvoxamine; HR, hazard ratio; Hyd, hydroxychloroquine; ICER, incremental cost-effectiveness ratio; ICU, intensive care unit; IF, interferon beta-1a; L + R, lopinavir + ritonivir; Mol, molnupiravir; N + R, nirmatrelvir + ritonavir; NR, not reported; QALY, quality-adjusted life-year; RCT, randomised controlled trial; Rt, disease reproduction rate; R + D, remdesivir and dexamethasone; SoC, standard of care; Sot, sotrovimab; SWQ, south-west quadrant (of the cost-effectiveness plane, i.e., lower cost and lower effectiveness); Toc, tocilizumab.

Notes: (Elvidge et al., 2022) This study (Ruggeri et al., 2022b) reports the cost results for “static” and “decreasing” infection rate scenarios the other way around, such that the cost in the “static” scenario is lower than the cost under decreasing infection rates. This appears to be an error, therefore we have swapped the cost results.

*Cost conversions to USD listed below. The OECD exchange rate for the reported price year is used (Exchange rates indicator, 2023). Where no price-year is explicitly reported, we have assumed the relevant exchange rate is the year prior to the year of publication.

• Alamer 2023 (Alamer et al., 2023): 1 USD = 3.750 SAR (2020).

• Lau 2022 (Lau et al., 2022): 1 USD = 1.341 CAD (2020).

• Metry 2022 (Metry et al., 2022), Rafia 2022 (Rafia et al., 2022): 1 USD = 0.727 GBP (2021).

• Ruggeri 2022 (Ruggeri et al., 2022a): 1 USD = 0.845 EUR (2021).

• Ruggeri 2023 (Ruggeri et al., 2023): 1 USD = 0.950 EUR (2022).

## 3 Results

### 3.1 Included studies

**FIGURE 1:**
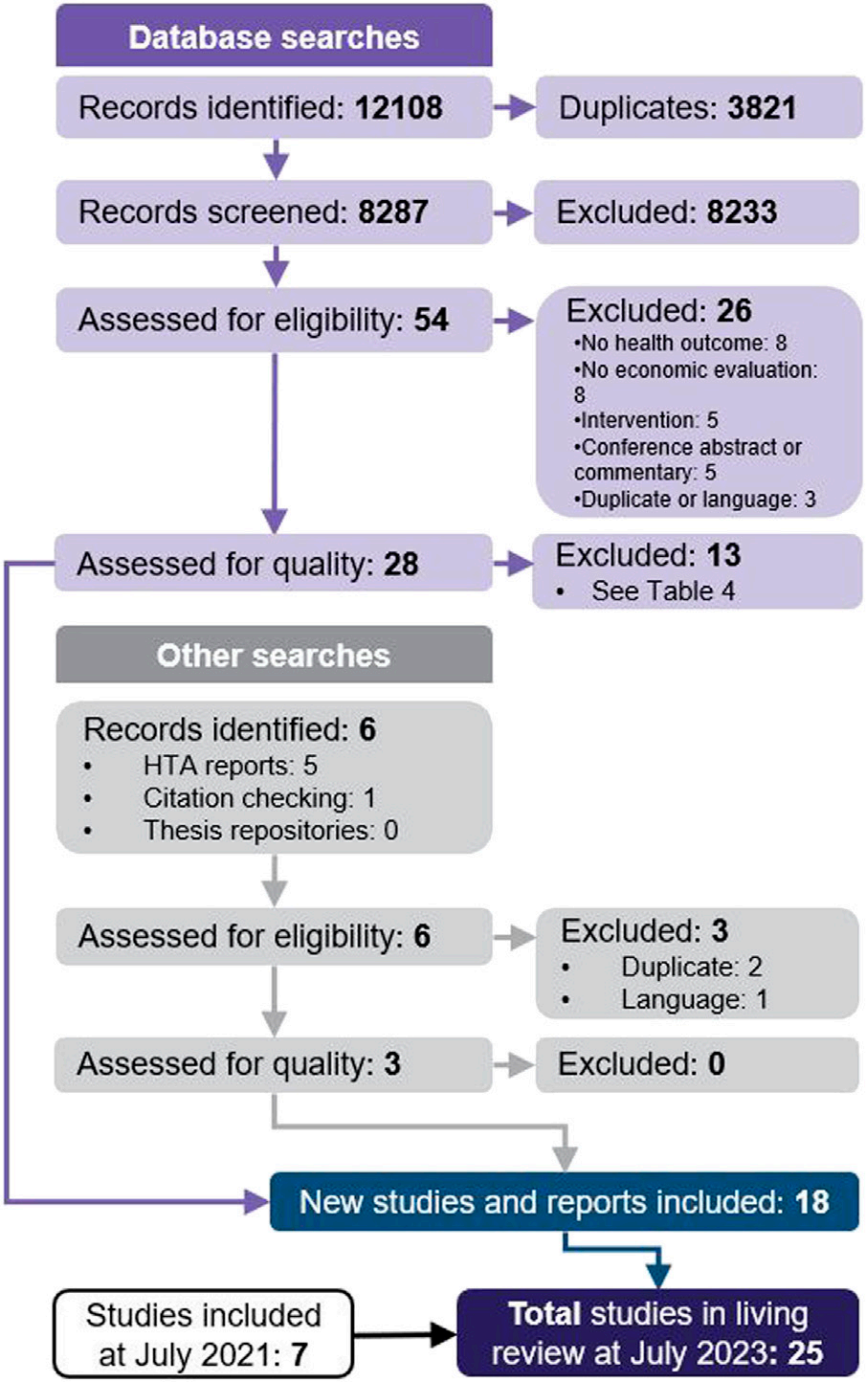
PRISMA diagram for study selection.

Search strategies and results per database are provided in Supplementary Material. A total of 8,287 unique records were identified for initial screening of titles and abstracts (Figure 1). Of those, 8,233 were excluded, most commonly because they did not report a primary economic evaluation. Therefore, 54 studies proceeded to full-text review, with 28 meeting the inclusion criteria. Six studies were also identified through searches of grey literature: 1 through citation checking, which met our inclusion criteria, and 5 HTA reports, of which 2 met our criteria. Two reported on the same HTA and were considered to be duplicates, and 1 was not available in English. A total of 31 studies proceeded to quality assessment, of which 13 were excluded due to the presence of very serious limitations (Table 4). Finally, 18 studies of acceptable quality were included in this two-year update (Carta and Conversano, 2021; Congly et al., 2021; Ruggeri et al., 2022a; Ruggeri et al., 2022b; Dijk et al., 2022; Goswami et al., 2022; Kelton et al., 2022; Lau et al., 2022; Metry et al., 2022; Park et al., 2022; Rafia et al., 2022; Savinkina et al., 2022; Yeung et al., 2022; Alamer et al., 2023; Arwah et al., 2023; Kowal et al., 2023; Ruggeri et al., 2023; Shah et al., 2023).

**TABLE 4:** Studies excluded due to having very serious limitations.

Study	Comparators & type of evaluation	Summary of very serious limitations that affect reliability of study conclusions
Beshah 2023 (Beshah et al., 2023)	Non-invasive ventilation, mechanical ventilation	Lifetime horizon, but no long-term outcomes included. Insufficient information about source of baseline outcomes. Relative effectiveness and resource use inputs sourced from a single non-randomised study without adjustment for selection bias. Limited analysis of uncertainty
	CUA	
Chow 2022 (Chow et al., 2022)	Statins, SoC	Health outcome is not defined and cannot be accurately inferred from the information reported. Relative effectiveness and resource use inputs sourced from non-randomised studies only. Unit costs are informed by World Health Organisation cost codes, generalisability to the study setting is unclear. No analysis of uncertainty reported
	CUA	
Gandjour 2022 (Gandjour, 2022)	Off-the-shelf self tests, personal protective measures + no testing	The design and appropriateness of the model structure are unclear (no schematic). Downstream effects of test results are not considered. Only costs associated with the test appear to have been included, and the unit price source is not reported. An ICER is reported, but the component incremental costs and QALYs are not. Many parameters are not subjected to uncertainty analysis
	CEA	
Jovanoski 2022 (Jovanoski et al., 2022)	Casirivimab + imdevimad, SoC	Some modelling assumptions and cost data sources may overstate the impact of the intervention. ICERs are reported, but the component incremental costs and QALYs are not. Minimal analysis of uncertainty is reported. There is a potential conflict of interest
	CUA	
Kilcoyne 2022 (Kilcoyne et al., 2022a)	Lezilumab, SoC	Time horizon (28 days) is too short to capture all relevant cost and health outcomes. Hospitalisation costs are sourced from a modelling study, when national schedules of costs are available. Estimates of clinical effectiveness are drawn from a single manufacturer funded RCT. Absolute values of intervention effect are used, which imposes the assumption of independent prior distributions in the treatment and comparator arms, which is unlikely. A joint measure of cost-effectiveness is not presented. A probabilistic sensitivity analysis is not performed. There is a potential conflict of interest
	CEA.	
Kilcoyne 2022 (Kilcoyne et al., 2022b)	Lezilumab, SoC	Time horizon (28 days) is too short to capture all relevant cost and health outcomes, though a scenario analysis extends the time horizon to 1 year. Absolute values of intervention effect are used, which imposes the assumption of independent prior distributions in the treatment and comparator arms. A joint measure of cost-effectiveness is not presented. A probabilistic sensitivity analysis is not performed. There is a potential conflict of interest
	CEA.	
Krylova 2021 (Krylova et al., 2021)	Favipiravir, umifenovir	No time horizon; clinical outcomes are directly dependent on the source of effectiveness evidence used (17 or 28 days). No long-term or downstream outcomes included. For one comparison, different RCTs are used to inform effectiveness, without adjustment for potential confounding. Substantial use of assumptions to inform resource use parameters. No uncertainty analysis
	CEA	
Ohsfeld 2021 (Ohsfeldt et al., 2021)	Baricitinib, SoC	Absolute values of intervention effect are used, which imposes the assumption of independent prior distributions in the treatment and comparator arms. Extensive use of unpublished “data on file”, assumptions and other various sources to inform resources use and cost parameters. Limited justification for ranges used in sensitivity analysis. There is a potential conflict of interest
	CEA, CUA.	
Oksuz 2021 (Oksuz et al., 2021)	Remdesivir, SoC	Time horizon (“a COVID-19 episode”) is unclear but is likely to be too short to capture all relevant cost and health outcomes. Baseline, relative effectiveness and resource use outcomes from unadjusted real-world data (*n* = 78). PSA distributions are not reported. There is a potential conflict of interest
	CUA	
Petrov 2022 (Petrov et al., 2022)	Anakinra, baricitinib, kanakinumab, levilimab, olokizumab, netakimab, sarilumab, secukinumab, tocilizumab, tofacitinib	The cited clinical evidence is insufficient to justify the assumption of equal effectiveness required by the chosen cost-minimisation approach. It is inappropriate to compare the studied interventions because are intended for different patient populations
	CBA	
Schallner 2022 (Schallner et al., 2022)	Intensive care, non-intensive care	Baseline outcomes for the standard care comparator arm (instant death) were arbitrary researcher assumptions, with no support from experts or data reported. Relative effectiveness inputs sources from a single, small study with a historical control. Costs associated with standard care not considered (instand death would not be costless). Limited analysis of uncertainty reported
	CUA	
Subhi 2023 (Subhi et al., 2023)	Remdesivir, favipiravir, SoC	No long-term outcomes are included. Source of baseline outcomes appears to be a trial conducted in a different setting. Relative effectiveness estimates for favipiravir rely on researcher assumptions. Indirect comparison between remdesivir and favipiravir is a naïve comparison. Resource use inputs informed by expert elicitation. Details of the experts and elicitation process are not reported. There is a potential conflict of interest
	CEA	
Wai 2023 (Wai et al., 2023)	Molnupiravir, nirmatrelvir + ritonavir, SoC	Time horizon (28 days) is too short to capture all relevant cost and health outcomes. Baseline outcomes and relative effectiveness outcomes sources from a single non-randomised study. Unclear how resource use inputs were recorded and how cost inputs were sourced. No analysis of uncertainty reported
	CEA	

Abbreviations: CBA, cost-benefit analysis; CEA, cost-effectiveness analysis; CUA, cost—utility analysis; ICER, incremental cost-effectiveness ratio; QALY, quality-adjusted life-year; RCT, randomised controlled trial; SoC, standard of care.

Included studies evaluated interventions in community or outpatient settings (5/18) (Goswami et al., 2022; Park et al., 2022; Savinkina et al., 2022; Yeung et al., 2022; Ruggeri et al., 2023), where patients are at risk of admission to hospital, or an inpatient hospital setting (11/18) (Carta and Conversano, 2021; Congly et al., 2021; Ruggeri et al., 2022a; Ruggeri et al., 2022b; Dijk et al., 2022; Kelton et al., 2022; Lau et al., 2022; Rafia et al., 2022; Alamer et al., 2023; Kowal et al., 2023; Shah et al., 2023); one study included both settings (1/18) (Metry et al., 2022). One study evaluated point-of-care tests in an unspecified health facility (1/18) (Arwah et al., 2023). Of studies based in inpatient hospital settings, some were aimed at specific populations and places within the care pathway, namely moderate disease with non-invasive ventilation (3/12) (Ruggeri et al., 2022a; Ruggeri et al., 2022b; Rafia et al., 2022) or critical care (1/12) (Shah et al., 2023), though most had mixed or unspecified populations (8/12) (Carta and Conversano, 2021; Congly et al., 2021; Dijk et al., 2022; Kelton et al., 2022; Lau et al., 2022; Metry et al., 2022; Alamer et al., 2023; Kowal et al., 2023). Most studies (12/18) (Carta and Conversano, 2021; Congly et al., 2021; Dijk et al., 2022; Goswami et al., 2022; Lau et al., 2022; Metry et al., 2022; Rafia et al., 2022; Savinkina et al., 2022; Yeung et al., 2022; Alamer et al., 2023; Kowal et al., 2023; Ruggeri et al., 2023) took a healthcare system or payer perspective in their base-case analyses, while 2/18 took a provider (e.g., hospital) perspective (Ruggeri et al., 2022a; Shah et al., 2023), 2/18 took a partial societal perspective (Kelton et al., 2022; Arwah et al., 2023), and 2/18 did not explicitly report a perspective (Ruggeri et al., 2022b; Park et al., 2022). Multiple studies were conducted in the United States (8/18) (Carta and Conversano, 2021; Congly et al., 2021; Dijk et al., 2022; Goswami et al., 2022; Kelton et al., 2022; Savinkina et al., 2022; Yeung et al., 2022; Kowal et al., 2023), Saudi Arabia (2/18) (Ruggeri et al., 2022b; Alamer et al., 2023) and the United Kingdom (2/18) (Metry et al., 2022; Rafia et al., 2022), while single studies were conducted in each of Canada (Lau et al., 2022), Italy (Ruggeri et al., 2023), Kenya (Arwah et al., 2023), Portugal (Ruggeri et al., 2022a), Singapore (Park et al., 2022) and Tanzania (Shah et al., 2023). Most studies reported costs in US dollars (12/18), with 4/18 converting to US dollars from the local currency (Ruggeri et al., 2022b; Park et al., 2022; Arwah et al., 2023; Shah et al., 2023), while 6/18 reported costs in the local non-US currency (Ruggeri et al., 2022a; Lau et al., 2022; Metry et al., 2022; Rafia et al., 2022; Alamer et al., 2023; Ruggeri et al., 2023).

Included studies evaluated one or more of the following pharmacological treatments for COVID-19, usually in addition to standard care: remdesivir (9/18) (Carta and Conversano, 2021; Congly et al., 2021; Ruggeri et al., 2022a; Ruggeri et al., 2022b; Dijk et al., 2022; Kelton et al., 2022; Lau et al., 2022; Metry et al., 2022; Rafia et al., 2022), casirivimab + imdevimab (3/18) (Dijk et al., 2022; Park et al., 2022; Ruggeri et al., 2023), baricitinib + remdesivir (3/18) (Dijk et al., 2022; Kelton et al., 2022; Metry et al., 2022), dexamethasone (3/18) (Carta and Conversano, 2021; Congly et al., 2021; Dijk et al., 2022), nirmatrelivir + ritonivir (3/18) (Metry et al., 2022; Savinkina et al., 2022; Yeung et al., 2022), molnupiravir (2/18) (Goswami et al., 2022; Yeung et al., 2022), and tocilizumab (2/18) (Dijk et al., 2022; Metry et al., 2022). The following medicines were evaluated by single studies: baricitinib (Metry et al., 2022), favipiravir (Alamer et al., 2023), fluvoxamine (Yeung et al., 2022), hydroxychloroquine (Dijk et al., 2022), interferon beta-1a (Dijk et al., 2022), lopinavir + ritonivir (Dijk et al., 2022), remdesivir + dexamethasone (Carta and Conversano, 2021), sotrovimab (Metry et al., 2022). One study (1/18) evaluated lenzilumab alongside other treatments (lenzilumab, molnupiravir and casirivimab + imdevimab) but did not publish cost-effectiveness results for these other treatments due to confidentiality (Metry et al., 2022). One study (1/18) evaluated a hypothetical pharmacological treatment for COVID-19, with an efficacy profile derived from the ACTT-1 (remdesivir) and RECOVERY (dexamethasone) trials, and a price of $2,500 per course (Kowal et al., 2023). In all cases, standard care without the pharmacological intervention of interest was a comparator. One study (1/18) evaluated a test for SARS-CoV-2 (Arwah et al., 2023), and one (1/18) evaluated the cost effectiveness of different levels of critical care for the treatment of severe COVID-19 in a lower-middle income country setting (Shah et al., 2023). The comparators were not treating COVID-19 in critical care services; treating COVID-19 with basic critical care in district hospitals, reflecting standard care; essential critical care, defined as treating people with severe and critical disease with advanced care such as supplemental oxygen; and advanced critical care, where people with critical disease are treated with life-sustaining therapies such as mechanical ventilation.

Cost—utility analyses (CUAs) were reported by 12/18 studies, quantifying costs and a preference-based measure of health (Carta and Conversano, 2021; Congly et al., 2021; Dijk et al., 2022; Goswami et al., 2022; Kelton et al., 2022; Metry et al., 2022; Park et al., 2022; Yeung et al., 2022; Arwah et al., 2023; Kowal et al., 2023; Shah et al., 2023). In most cases (9/12), quality-adjusted life years (QALYs) were used (Carta and Conversano, 2021; Congly et al., 2021; Dijk et al., 2022; Goswami et al., 2022; Kelton et al., 2022; Metry et al., 2022; Rafia et al., 2022; Yeung et al., 2022; Kowal et al., 2023); the rest (3/12) used disability-adjusted life years (DALYs) (Park et al., 2022; Arwah et al., 2023; Shah et al., 2023). One of the QALY-based analyses was a *distributional* CUA (Kowal et al., 2023), and was a re-analysis of a study that was included in the initial review (Sheinson et al., 2021). Cost-effectiveness analyses (CEAs) were reported by 7/18 studies (Ruggeri et al., 2022a; Ruggeri et al., 2022b; Lau et al., 2022; Park et al., 2022; Savinkina et al., 2022; Alamer et al., 2023; Ruggeri et al., 2023). All CEA studies used deaths averted as their non-preference-based measure of health. One study (1/18) was a CEA conducted alongside a clinical trial (Lau et al., 2022). All other studies (17/18) reported model-based analyses, comprising decision trees (6/17) (Carta and Conversano, 2021; Congly et al., 2021; Metry et al., 2022; Park et al., 2022; Savinkina et al., 2022; Arwah et al., 2023); Markov models (6/17) (Ruggeri et al., 2022a; Ruggeri et al., 2022b; Dijk et al., 2022; Kowal et al., 2023; Ruggeri et al., 2023; Shah et al., 2023), some of which were nested within a disease epidemiology model (3/17) (Ruggeri et al., 2022a; Ruggeri et al., 2022b; Ruggeri et al., 2023); “hybrid” models, with a decision tree to model acute disease followed by a long-term Markov component (3/17) (Goswami et al., 2022; Kelton et al., 2022; Yeung et al., 2022); partitioned survival models (2/17) (Metry et al., 2022; Rafia et al., 2022); and a patient-level simulation (1/17) (Alamer et al., 2023). A potential financial conflict of interest in favour the intervention under evaluation was reported by 6/18 included studies (Ruggeri et al., 2022b; Goswami et al., 2022; Kelton et al., 2022; Lau et al., 2022; Ruggeri et al., 2023).

### 3.2 Cost effectiveness

#### 3.2.1 Treatments: inpatient hospital setting

For evaluations based in inpatient hospital populations, with or without supplemental oxygen, 8/12 studies were CUAs that specified one or more willingness-to-pay thresholds to determine cost effectiveness of evaluated interventions. Dexamethasone was found to be cost effective compared with standard care (Carta and Conversano, 2021; Congly et al., 2021; Dijk et al., 2022); this conclusion was robust to sensitivity analyses, and a value of information analysis indicated there would be no value in further research (Dijk et al., 2022). Remdesivir was also generally cost effective *versus* standard care (Carta and Conversano, 2021; Congly et al., 2021; Dijk et al., 2022; Rafia et al., 2022), though 1 study noted that this result was highly sensitive to whether it confers a survival benefit or not (Rafia et al., 2022). If it does, the reported ICER was around $17,000 per QALY gained, rising to over $1 million per QALY gained if it does not reduce the risk of death. Its mortality hazard ratio should be at least 0.92 for it to be cost effective. Further, 1 study found that using dexamethasone for all hospitalised patients dominated any strategy that involved remdesivir (Congly et al., 2021). Baricitinib in addition to remdesivir was found to be cost effective *versus* standard care in 2 studies (Dijk et al., 2022; Metry et al., 2022), and this was robust to sensitivity analyses. In a US study (Kelton et al., 2022), it had an ICER of around $22,000 per QALY gained compared with using remdesivir alone, in a partial societal analysis, and was dominant from a hospital perspective. However, this was in conflict with a fully incremental analysis from a United Kingdom healthcare perspective (Metry et al., 2022), that suggested barcitinib monotherapy was the most cost effective treatment for hospitalised patients, with ICERs of around $7,500–19,000 per QALY gained depending on the patient’s need for oxygen. The conflicting results may be explained by healthcare resource cost differences between the US and United Kingdom, or the studies’ different sources for relative effectiveness data; one (Kelton et al., 2022) made use of data on file from a single trial (ACTT-2), while the other (Metry et al., 2022) used outputs from published “living” network meta analyses (metaEvidence Initiative, 2022; The COVID-NMA Initiative, 2021). In a study that compared treatments with standard care but not with each other (Dijk et al., 2022), casirivimab + imdevimab and tocilizumab were estimated to have ICERs of around $13,000 and $40,000 per QALY gained, respectively, while hydroxychloroquine, interferon beta-1a and lopinavir + ritonivir were found to be cost saving but detrimental to health outcomes (QALYs). The resulting southwest-qaudrant ICERs were around $46,000, $5,500 and $15,000, respectively, which, at the specified threshold of $100,000 per QALY gained, imply the cost savings would not be sufficient to offset the health outcomes foregone. Value of information analysis identified that further research to examine disinvestment in hydroxychloroquine may be worthwhile, though it is not widely used.

One QALY-based evaluation of inpatient pharmacological treatment (Kowal et al., 2023) was a distributional re-analysis of a study previously included in this review (Sheinson et al., 2021). Treatment was found to be more cost effective in more deprived populations, with an ICER of $28,000 per QALY gained in the most deprived group and $29,800 in the least deprived group. Including the existing inequitable distribution of opportunity costs in the US health system, population-level net health benefits would remain positive up to a treatment cost of $60,100 per patient.

The one other CUA in the inpatient setting (Shah et al., 2023) evaluated using different levels of critical care to treat people with COVID-19, and used DALYs averted as the health outcome. At a specified conservative threshold in Tanzania of $101 per DALY averted, using essential critical care (e.g., supplemental oxygen) for people with COVID-19 was cost effective compared with no critical care, with an ICER of $37 per DALY averted, and district-level critical care ($14 per DALY averted). The equivalent ICERs for using advanced critical care (e.g., mechanical ventilation critical disease) were above the threshold, at $186 and $144, respectively.

There were 4 CEAs evaluating treatments in the inpatient setting, with all using deaths averted as the health outcome. Two studies used the same economic model with country-specific input data, and found remdesivir to reduce deaths and costs compared with standard care in Portugal [7 deaths averted and $1.2 m saved per 1,000 cases (Ruggeri et al., 2022a)] and Saudi Arabia [6.7, $980,000 (Ruggeri et al., 2022b)]. One trial-based analysis reached similar results in the setting of Canada (Lau et al., 2022); it was dominant in 58% of probabilistic sensitivity analysis (PSA) simulations, and the ICER was below $100,000 for death averted in 82%. Favipiravir was evaluated by 1 study (Alamer et al., 2023), and was also found to reduce deaths and costs in Saudi Arabia, with a saving of $4,500 per death averted.

#### 3.2.2 Treatments: outpatient and community setting

For patients treated in the outpatient and community setting, at risk of progressing to severe disease requiring inpatient or critical care, 4 studies compared active treatments with standard care only. Among them, casirivimab + imdevimab was found to have an ICER of $800 per DALY averted in 1 study (Park et al., 2022)–below the specified $74,000 per DALY threshold in Singapore–and to reduce deaths and save costs in Italy in another study (Ruggeri et al., 2023). The conclusions of both studies were robust to sensitivity analyses undertaken; in the latter case, casirivimab + imdevimab was dominant in over 90% of simulations. One study estimated that molnupiravir would dominate standard care, generating incremental QALYs and reducing costs (Goswami et al., 2022).

In the only identified study that explicitly compared different strategies for using a treatment in subgroups defined by vaccination status (Savinkina et al., 2022), base-case results suggested that nirmatrelivir + ritonivir would dominate standard care for unvaccinated high-risk groups. However, that result was sensitive to the relative effect estimate, with a plausible lower-bound effect leading to an ICER of over $300,000 per death averted. Nirmatrelivir + ritonivir would be less cost effective if used in vaccinated and low-risk groups, with the ICER rising to $5 m per death averted if used for all patients. In a US HTA analysis (Yeung et al., 2022), it had an ICER of $21–26,000 per QALY gained *versus* standard care, depending on the perspective chosen, and in a United Kingdom fully incremental analysis conducted for HTA (Metry et al., 2022), the ICER was around $8,500 per QALY gained (remdesivir and sotrovimab were dominated). The US HTA study also found fluvoxamine would be cost effective compared with standard care relative to typical US thresholds (ICER: $8–20,000 per QALY gained), while molnupiravir had an ICER of $61,000 per QALY gained from a healthcare perspective, and $43,000 per QALY gained from a partial societal perspective that captured productivity costs. Therefore, the perspective would be a relevant factor for healthcare decision makers using the specified conservative threshold of $50,000 per QALY. In a scenario focusing on an unvaccinated patient population, in whom the effects of COVID-19 may be more severe, the US HTA (Yeung et al., 2022) found that nirmatrelivir + ritonivir, fluvoxamine and molnpiravir would be more cost effective *versus* standard care (with healthcare perspective ICERs of $15,000, $4,000 and $48,000 per QALY gained, respectively).

#### 3.2.3 Diagnostic tests

The single study that evaluated a diagnostic test (Arwah et al., 2023) found that rapid antigen tests, plus a delayed nucleic acid amplifying test (NAAT) used in a confirmatory way, had an ICER of $965 per DALY averted compared with typical standard care in Kenya: delayed NAAT alone. This would be cost effective by a close margin relative to the specified local threshold of $1,003 per DALY; in PSA, the probability of the ICER being below the threshold was 53%. The ICER was sensitive to the underlying disease prevalence and the accuracy of rapid and confirmatory tests. In a scenario where NAAT is not available, rapid testing was estimated to dominate a “no testing” strategy that relies on clinical judgement.

## 4 Discussion

### 4.1 Principal findings

This updated systematic review indicates that pharmacological treatments that have been repurposed for to treat COVID-19 in recent years have the potential to be cost effective. In particular, the use of the low-cost corticosteroid dexamethasone—which has become routine practice to treat severe COVID-19 in an inpatient setting—appears to be clearly cost effective. Remdesivir and baricitinib, potentially in combination, appear to be promising candidates to treat severe disease, too. Limited cost-effectiveness evidence in the inpatient setting for casirivimab + imdevimab, tocilizumab, hydroxychloroquine, interferon beta-1a and lopinavir + ritonivir suggests the latter 3 treatments may produce worse health outcomes than standard care without a commensurate cost saving to be considered by decision makers.

In the outpatient and community setting, there is some evidence that casirivimab + imdevimab, fluvoxamine and molnupiravir may be cost effective over standard care. Results from 3 studies indicate that nirmatrelivir + ritonivir may be a cost effective treatment in the community setting among patients at high risk of hospitalisation (such as unvaccinated people), though the one fully incremental analysis among them does not include casirivimab + imdevimab, fluvoxamine or molnupiravir in the published results. The 2 studies that evaluated non-pharmacological interventions were both in lower-income settings. They suggested that rapid antigen tests may be cost effective where there is slow existing testing infrastructure, and certainly where there is none; and using the most advanced forms of critical care to treat COVID-19 might be difficult to justify, on cost-effectiveness grounds, in a resource-limited setting.

Compared with the first iteration of this review (Elvidge et al., 2022) conducted in July 2021, this update has identified economic evaluations of a much larger set of interventions for COVID-19. Previously, the evidence base was limited to evaluations of monotherapy and combination therapy use of dexamethasone and remdesivir, which were early candidate interventions for the treatment of COVID-19 in hospital. While we have identified additional cost-effectiveness evidence regarding these treatments, other pharmacological interventions have received marketing authorisation to treat the disease since 2021, and it is logical that healthcare decisions makers will be interested in understanding which of them offer value for money. Here, we have identified such evidence for antiviral therapies (casirivimab + imdevimab, favipiravir, lopinavir + ritonivir, molnupiravir, nirmatrelivir + ritonivir), immunotherapies (baricitinib, sotrovimab, tocilizumab) and various other repurposed medicines (fluvoxamine, hydroxychloroquine, interferon beta-1a).

We identified 1 study evaluating the cost effectiveness of a test for SARS-CoV-2, representing 6% of our included studies (1/18) compared with 29% in the initial review (2/7). It is likely that this reflects the changing pandemic context over time, such that comparing alternative testing strategies is no longer a prominent concern, relative to assessing the value of the growing number of available treatment options. The study evaluating rapid antigen tests was one of 2 included studies evaluating non-pharmacological interventions; the other estimated the value of using scarce critical care resources to treat people with COVID-19. Notably, both studies were conducted in lower-income settings (Kenya and Tanzania, respectively). This suggests testing strategies and the allocation of scarce hospital resources for COVID-19 remain a prominent concern for healthcare decision makers in settings where the most effective and newly licensed pharmacological interventions are less likely to be available.

The context around COVID-19 has changed substantially since the first iteration of this review. However, it does not appear that economic evaluations have adapted their methods to reflect these changes. This is likely to limit their scope to inform decision-making. First, vaccination programmes have been successful across the world and the vast majority of people, particularly in high and middle income countries, have now received at least one dose of a vaccine (Mathieu et al., 2020). Despite this, there appears to be limited consideration within economic evaluations of the impact of vaccination on disease severity and implications for cost effectiveness. Some identified studies did report scenario analyses in unvaccinated populations who are more likely to experience severe symptoms, and one (Savinkina et al., 2022) study from the US explicitly compared alternative strategies for using nirmatrelivir + ritonivir in different patient subgroups defined by their vaccination status and risk of hospitalisation. This approach is likely required to properly define who would benefit from treatments, but has not become widespread. Second, there are several reasons that parameters derived from studies completed at different stages of the pandemic may not be generalisable to the present day. These include pressures and constraints on hospital care during acute phases of the pandemic, differing approaches to standard care and changing disease variants. One study (Yeung et al., 2022) reported a scenario analysis in the context of a hypothethical variant with lower baseline risk of hospitalisation, though this did not consider differential treatment effectiveness for different variants. Differential efficacy between variants has been observed in practice, and means cost-effectiveness evaluations may need to increasingly examine value for money in specific subpopulations (Coronavirus COVID-19 Update, 2022). While some studies did note that the changing composition of COVID-19 over time may limit the generalisability of their cost-effectiveness conclusions (Metry et al., 2022; Park et al., 2022; Ruggeri et al., 2023), this appears to be an underconsidered issue. Third, there are now treatments that are established as best-practice due to the emergence of results from large-scale platform trials. Indeed, within this review dexamethasone is highlighted as a low-cost option that is effective for patients with respiratory support. However, there have been limited attempts to compare established treatments with *each other*. There is a need for more fully incremental cost-effectiveness analyses that compare alternative options simultaneously, rather than indirectly through how they compare with standard care. This may require measures of relative effectiveness to be derived from network meta analyses, such as used Metry et al. (2022), rather than data from local sites or individual trials.

In terms of the analytical methods used, most included studies were model-based analyses, which is consistent with the first iteration of this review. The modelling methods used remained similar. Time horizons varied from short term to lifetime; decision tree, Markov, and hybrid model structures were prominent; the utility weights used by CUAs were often generalised from non-COVID sources; and the known long-terms effects of disease (“long COVID”) were generally not captured. However, it is notable that some models may be overrepresented in the evidence base, due to repeated adaptations or reanalyses of the same model. In particular, remdesivir was evaluated in settings of Portugal (Ruggeri et al., 2022a) and Saudia Arabia (Ruggeri et al., 2022b) using a common model with country-specific inputs; casirivimab + imdevimab was evaluated in Italy (Ruggeri et al., 2023) using essentially the same model; and an existing CUA of a hypothethical treatment was reanalysed through a distributional lens (Kowal et al., 2023).

### 4.2 Strengths and limitations

This update to our “living” systematic review is methodologically consistent with the first publication (Elvidge et al., 2022), and so the same issues concerning the search strategy and generalisability of findings apply here. Our review aimed to provide a comprehensive account of available evidence and as such, a large number of unique records were identified by the database search (8,287); this is due to the known sensitivity of search terms used to identify economic evaluations (Hubbard et al., 2022). This increases the sensitivity of the search, reducing the likelihood of missing relevant studies, but it also means future updates will continue to require a labour-intensive screening process. In addition, like before, we chose to exclude studies that met our selection criteria but were judged to have very serious limitations that may materially affect the conclusions about cost effectiveness. This follows the process used in NICE clinical guideline development (National Institute for Health and Care Excellence NICE, 2012), to avoid conflating results across studies of varying quality, and was predefined in our study protocol (Elvidge et al., 2021). In this update, it led to the exclusion of 13 potentially relevant studies (Table 4). While 2 reviewers conducted this quality assessment, discussing and resolving any disagreements, we recognise that this is necessarily subjective; other reviewers may have reached different quality assessment decisions, or simply included all studies that met the selection criteria. There may also be included studies which did not meet the bar for exclusion, but which have analytical flaws or are based on parameters that are biased or do not reflect best available evidence and this may impact their findings. Further, we excluded public health interventions (e.g., lockdowns, face masks) and vaccinations from our review. The economic value of such interventions may still be of interest to some healthcare decision makers, for example where vaccine coverage is relatively low. Finally, our review is, like any, at risk of publication bias. Several of the included studies exhibit a potential conflict of interest. We cannot know whether those analyses would have reached publication if they had demonstrated negative conclusions about the intervention, nor how many such analyses exist and were not published.

### 4.3 Implications for future research

After 3 years’ worth of cost-effectiveness research for COVID-19 interventions, there are some new and some persistent evidence gaps that would benefit from further thought. Head-to-head economic evaluations of interventions are in the minority of the identified studies. There are now several treatments available in both the pre-hospital and hospital settings, and fully incremental analyses that compare options simultaneously may be valuable for decision making. The cost effectiveness of tests and treatments may be influenced by what happens later in the clinical pathway, and so a whole-disease model that reports fully incremental results would be a valuable resource. In the context of a continuously evolving disease, and with variable standard care and vaccination uptake in different settings, a whole-disease model could allow for rapid re-analyses in light of new evidence or changes in the prevailing conditions. In general, researchers should routinely reflect on the potential implications of vaccination status and disease variants for the generalisability of their conclusions. Further, these authors recommend that researchers routinely conduct detailed sensitivity analyses examining potential cost effectiveness under a wide range of baseline outcomes and relative effectiveness. Such analyses may help to ‘future-proof’ their studies to ensure they remain useful under different prevailing coditions. There remains a need for robust evidence about the health-related quality of life impact of COVID-19 in the short and long term, to support the conduct of CUAs. Finally, several identified studies were CEAs that used deaths averted as their health outcome with relatively short time horizons. This may be reasonable in some circumstances, and particularly if one intervention appears both more effective and have lower costs. However, in general, decision makers should be aware that where a treatment has an effect on survival, short-term analyses will not fully capture all relevant differences in outcomes between it the comparator.

## 5 Conclusion

This updated review of economic evaluations of tests and treatments for COVID-19, covering the period from July 2021 and July 2023, provides a contemporary summary of the cost-effectiveness evidence. Compared with the first iteration of the review (up to July 2021), we have identified 18 additional studies of acceptable quality that healthcare decision makers, such as HTA and payer organisations, may consider to inform their COVID-related decision making. In particular, the evidence may support prioritisation between the numerous antiviral therapies and immunotherapies. Conclusions about some treatments, such as dexamethasone (cost effective) and hydroxychloroquine (not cost effective), support current standard practice. An evidence gap remains for a whole-disease model that can support holistic decision making about multiple tests and treatments at linked decision points in a fully incremental way.

## Data Availability

The original contributions presented in the study are included in the article/Supplementary Material, further inquiries can be directed to the corresponding author.
