# Supplementary material for: Diagnostics and treatments of COVID-19: two-year update to a living systematic review of economic evaluations
Source: Front Pharmacol. 2023 Nov 16;14:1291164. doi: 10.3389/fphar.2023.1291164 (PMC10687367; doi:10.3389/fphar.2023.1291164)
Supplement: Supplementary file 1 [file Table1.docx]

Supplementary Table 1. Results of included studies

| **Study** | **Cost and health outcome results** | **ICER/net benefit of intervention(s) vs. comparator(s)** | **Cost-effectiveness threshold (if relevant)** | **Sensitivity & scenario analyses** | **Authors’ conclusions regarding cost effectiveness** | **Authors’ reported limitations and challenges** |
| --- | --- | --- | --- | --- | --- | --- |
| Alamer 2023 (14) | Favipiravir: $17,197*; 0.97 probability survival  SoC: $35,331*; 0.93 probability survival | -$4,534* per death averted | No threshold reported  Report no agreed thresholds in Saudi but dominant | Analysis replicated with weighted model using propensity scores and PSA completed for weight and unweighted analyses.  Favipiravir is less costly and more effective across all analyses | Favipiravir was associated with lower cost than SoC in both the unweighted and the weighted models. Favipiravir was also associated with a higher probability to be discharged alive | Limiting to deaths averted and time to discharge may miss important outcomes. Extending to CUA was not possible but would be desirable.  Study did not explore timing of treatment which may be important |
| Arwah 2023 (15) | Scenario 1 (access to confirmatory testing)  Rapid testing with delayed confimatory testing: $1,336,231, 1999 DALYs  Delayed testing: $1,107,118, 2236 DALYs  Scenario 2 (no access to confirmatory testing)  Rapid testing: $998,260.67, 2538 DALYs  Clinical judgement: $1,261,230, 2361 DALYs | Scenario 1  $965 per DALY averted (rapid testing more costly and more effective)  Scenario 2  $1490 per DALY averted (clinical judgement more costly and more effective) | $1003 (stated as Kenyan threshold) | Deterministic sensitivity with key parameters varied and PSA.  Cost-effectiveness was sensitive to changes in the prevalence, changes to sensitivity and specificity of rapid testing and confirmatory testing  Scenario 1:  Rapid testing had probability of 52.5% of being cost-effective at threshold.  Scenario 2:  Rapid testing had probability of 71.% of being cost-effective at threshold | Using rapid testing as a first-line tool, and later confirmatory tests of negatives where available, was a cost-effective strategy. Otherwise, rapid testing is preferred to clinical judgement, although it is less costly and less effective. | Limited by unavailable data on outcomes for false negative patients |
| Carter & Conversano 2021 (12) | SoC: $33,370, 0.767 QALYs  Remdesivir: $32,354, 0.773 QALYs  Dexamethasone: $33,556, 0.803 QALYs  R+D: $32,540, 0.809 QALYs | R+D dominates both SoC and dexamethasone. R+D vs. remdesivir: $5,222/QALY.  Excluding R+D:  Remdesivir dominates SoC.  Dexamethasone vs remdesivir: $40.6K/QALY. | $50K/QALY gained | OWSA results presented vs. SoC only. All ICERs vs. SoC robust except when remdesivir relative effect takes lower bound estimate (R+D: $24.4K/QALY, remdesivir: $261K/QALY). PSA results consistent with base case. | This analysis supports the use of remdesivir and/or dexamethasone. | Analysis is based on limited evidence of treatment effectiveness. R+D does not have proven effectiveness. Disease progression, long-COVID and different patient characteristics not explored. Proxy utility data used. |
| Congly 2021 (11) | Total costs & QALYs  (Strategies denoted by treatment for moderate disease, treatment for severe disease.)  1. SoC, SoC: $11.1K, 0.716  2. SoC, Dex: $11.1K, 0.726  3. Dex, Dex: $11.1K, 0.735  4. SoC, Rem: $11.8K, 0.710  5. Rem, SoC: $13.1K, 0.725  6. Rem, Dex: $13.1K, 0.734  7. Rem, Rem: $13.7K, 0.719 | All ICERs for remdesivir strategies are dominated by giving dexamethasone to all (moderate and severe) patients.  Dexamethasone for severe only, vs. SoC: $285/QALY.  Dexamethasone for all patients vs. severe only: $1,718/QALY. | $100K/QALY gained | Optimal strategy is not sensitive to OWSA.  PSA: ICER for dexamethasone (all patients) is below £100K/QALY with 98% probability. | Dexamethasone for all patients was the most cost-effective strategy. Dexamethasone for severe cases would be favoured at lower decision thresholds. | Analysis is based on limited evidence of treatment effectiveness. Fixed DRG costs do not account for different hospital stay durations. No long-term health outcomes. Proxy utility data used. |
| Dijk 2022 (5) | Incremental vs. SoC  Hydroxychloroquine (Hyd): -$12,227, -0.263 QALYs.  Remdesivir (Rem): -$5, +0.252 QALYs.  C+I: $696, +0.171 QALYs.  Dexamethasone (Dex): $6856, +0.614 QALYs.  B+R: $10,673, +0.775 QALYs.  Tocilizumab (Toc): $35,849, +0.882 QALYs.  Interferon b1 (IF): -$2,538, -0.472 QALYs.  L+R: -$1,404, -0.091 QALYs. | ICERs vs. SoC  Hyd: $46,427 (SWQ).  Rem: Dominant.  C+I: $4,075.  Dex: $11,619.  B+R: $13,772.  Toc: $40,633.  IF: $5,377 (SWQ).  L+R: $15,418 (SWQ).  Fully incremental NR due to heterogeneous SoC arms across trials. | $100K/QALY gained | Value of information analysis  Decisions about Dex, C+I, B+R, L+R and IF would not change with  further evidence.  For Rem and Toc, the value of further evidence would not outweigh the cost of research.  For Hyd, further evidence to investigate decremental cost effectiveness may be worthwhile. | At a threshold of $100K/QALY gained, treatment with remdesivir, C+I, dexamethasone, B+R and tocilizumab are cost effective versus SoC. | Some parameters were estimated from non-COVID studies. Effectiveness estimates drawn from single trials.  Analysis focuses on the research and approval health policy questions, not comparisons |
| Goswami 2022 (6) | Molnupiravir: $8,795, 17.721 QALYs  SoC: $9,690, 17.512 QALYs | Molnupiravir is dominant compared with SoC. | $100K per QALY gained | Results robust to scenario and one-way sensitivity analyses. PSA: Molnupiravir 100% likely to have an ICER below threshold. | Compared with SoC, treatment with molnupiravir can be considered a cost-effective option in the management of outpatients with COVID-19 at risk of progression to severe disease in the US. | Other outpatient treatments for COVID-19 not included. Appropriate utility data were unavailable and required primary research. |
| Kelton 2022 (13) | Partial societal perspective  Remdesivir: $372K, 11.7 QALYs.  B+R: $380K, 12.1 QALYs  Note: >80% of costs composed of other long-term medical costs.  Hospital perspective B+R vs. remdesivir: -$1,778, +0.0018 QALYs. | Partial societal perspective B+R vs. remdesivir: $22.3K/QALY, $17.9K/LYG.  Hospital perspective B+R dominates remdesivir. | $50K/QALY gained | All ICERs robust to OWSA, including oxygen/NIV subgroup. B+R more cost effective if no survival benefit (due to future unrelated medical costs avoided).  PSA: consistent with deterministic. | B+R is more cost effective than remdesivir alone for patients hospitalised because of COVID-19 in the US. | Lack of data to inform long-term burden of COVID-19.  Analysis does not capture potential readmissions or resource capacity constraints. Data informing utility values are limited.  National average DRG costs may lack generalisability. |
| Kowal 2023 (16) | Deterministic incremental results  Average:  Costs $12,741, QALYs +0.445 | Deterministic results  Average: $28,600 per QALY gained  Highest deprivation: $28,000 per QALY gained  Lowest deprivation: $29,800/QALY gained  Including inequitable opportunity costs (NHB)  Total: 735,569 QALYs  Hispanic, highest deprivation: 72,083; lowest deprivation: 1,106.  Black, highest deprivation: 47,342; lowest deprivation: 1,622.  White, highest deprivation: 113,982; lowest deprivation: 27,450. | $150,000 per QALY gained ($50K & $100K in sensitivity analyses) | Population NHB by threshold:  $50K: 391,114 QALYs.  $100K: 649,456 QALYs.  Population NHB remain positive up to inpatient treatment cost of $60,100 per patient. | Funding COVID-19 treatments reduced the population-level burden of health inequality by 0.234% (or 130,000 QALYs).  Distributional CUA of inpatient COVID-19 treatments may improve overall health while reducing health inqualities. | Underreporting of COVID-19 cases, hospitalisations deaths, and potentially variable reporting across equity subgroups. 20% of the population was not captured by the DCUA.  No trial data were identified that reported subgroup effects.  Lack of quality-of-life data at the subgroup level. |
| Lau 2022 (17) | Remdesivir: $28,276*, 0.809 deaths averted  Placebo: $28,357*, 0.771 deaths averted | Dominant | Thresholds of $0, $14,914*, $37,286* and $74,571* used for interpreting PSA | Results similar across deterministic scenarios. Major drivers of cost effectiveness were inpatient care and remdesivir costs.  Remdesivir dominant in 58% of PSA simulations and below $74,571* in 82%. | Remdesivir plus SoC is likely the preferred treatment strategy compared with usual care alone, for hospitalised adults with COVID-19. | Short time horizon may miss downstream costs and later events.  Data from RCT may not reflect routine clinical practice. |
| Metry 2022 (9) | Total costs* & QALYs  In hospital, on oxygen:  SoC: $30,436, 4.61  Toc: $35,146, 5.12  Rem: $38,202, 5.08  Baricitinib (Bar): $41,572, 5.46  B+R: $41,974, 5.32  In hospital, no oxygen:  SoC: $13,316, 5.79  Bar: $16,073, 6.29  Rem: $16,487, 6.07  B+R: $17,509, 6.21  In the community, high risk:  SoC: $1,448, 13.42  N+R: $2,483, 13.53  Sot: $4,924, 13.48  Rem: $6,039, 13.45 | In hospital, on oxygen:  SoC: reference  T: $9,254*  Rem: dominated  Bar: $18,812*  B+R: dominated  In hospital, no oxygen:  SoC: reference  Bar: $7,564*  Rem: dominated  B+R: dominated  In the community, high risk:  SoC: reference  N+R: $8,484*  Sot: dominated  Rem: dominated | $27.5K* /QALY gained | Treatments are more cost effective when duration of long COVID was shorter, and in younger patients. In the community setting, a higher risk of hospitalisation makes early treatment more cost effective. | In hospital, all treatments evaluated had scenarios where the ICER vs. SoC was below the threshold.  In the community setting, N+R may be cost effective compared with SoC. | The decision problem has evolved, so studies do not reflect the current conditions. Therefore, many assumptions were required. No head-to-head studies of interventions were identified. Confidential results not published for lenzilumab, molnupiravir or casirivimab + imdevimab. |
| Park 2022 (7) | Incremental results (costs and DALYs averted)  Treatment with C+I vs SoC:  Treat ages ≥80: -$0.08m, 38  Treat ages ≥70: -$0.1m, 66  Treat ages ≥60: -$0.34m, 161  Treat ages ≥50: +$0.17m, 198 | Treatment with C+I vs SoC:  Dominant  Dominant  Dominant  $800/DALY averted | 1.15 gross national income per DALY = $74K in 2021. | Results were robust to sensitivity analyses setting the relative risk reduction to the 95% CI bounds. | All strategies considered were cost effective using the specified threshold.  Treating people aged ≥60 was the most cost saving strategy. | Study prior to widespread circulation of delta and omicron disease variants. Efficacy and cost effectiveness of C+I may differ by variant. |
| Rafia 2022 (8) | Total costs* and QALYs (probabilistic)  If remdesivir has a survival effect:  SoC: $12,920, 6.35  Remdesivir: $17,549, 6.62  If remdesivir has no survival effect:  SoC: $14,190, 6.35  Remdesivir: $16,481, 6.35 | If remdesivir has a survival effect:  ICER vs. SoC: $17,056*.  If remdesivir has no survival effect:  ICER vs. SoC: >$1M. | $27.5K* /QALY gained | ICERs most affected by time horizon, baseline survival with SoC, and inclusion of unrelated costs. At analysis price, remdesivir mortality HR must be 0.915 or higher to be cost effective.  PSA: ICER below threshold with 74% probability if it confers a survival benefit, else 0%. | Remdesivir is likely to be cost effective only if it prevents death, and this is highly uncertain within the supplemental oxygen population. | Rapidly changing context means some parameter estimates and assumptions out of date.  Model cannot track individual patients.  Analyses conducted at list prices, may not reflect true prices paid.  Potentially some double counting of COVID-19 disutility. |
| Ruggeri 2022 (18) | Incremental results  23,579 cases:  Costs -$27.8m*  Deaths averted 165.9  Calibrated to 1,000 cases:  Costs -$1.2m*  Deaths averted 7.0 | NR | NR | Results sensitive to Rt; admission, ICU and mortality rates; remdesivir treatment effect. However, conclusions remain the same.  PSA: results not reported in detail, but remdesivir appears to be cost-incurring (i.e., not dominant) in a significant proportion of PSA results. | The ability of remdesivir to decrease ward LoS and ICU admissions would produce signifcant cost savings for hospitals, a more manageable hospital capacity in a public health emergency, and a faster recovery for hospitalised patients who require supplemental oxygen. | Infection forecasts were informed by various sources, including historical data and expert opinion, and are therefore uncertain. Potential side effects of remdesivir were not included. |
| Ruggeri 2022 (19) | Incremental results  Static infection rate (178,405 cases):  Costs -$174.81m  Deaths averted (DA) 1.2  Calibrated to 1,000 cases:  Costs -$979,836  Deaths averted 6.7 | NR | NR | Results sensitive to Rt values, ICU and mortality rates, baseline hospitalisation and remdesivir mortality effect, but conclusions remain the same.  Rt=0.8 (decreasing; 109,087 cases): costs -$154.7m^1^, DA 815.  Calibrated to 1,000 cases: costs -$1.4m, DA 7.5.  Rt=1.2 (increasing; 247,724 cases): costs -$377.3m, DA 1,582.  Calibrated to 1,000 cases: costs -$1.5m, DA 6.4.  PSA: remdesivir is dominant in 93% of simulations. | In Saudi Arabia, remdesivir plus standard of care has the potential to reduce healthcare resource use, mortality, and costs when compared with  standard of care alone across a range of plausible local epidemiological scenarios. | Some infection forecasts were informed by expert opinion, and are therefore uncertain. Many inputs informed by targeted, rather than systematic, literature review, including only 1 RCT. Treatment-related adverse eventsnot captured. |
| Ruggeri 2023 (22) | Incremental results  194,451 cases:  Costs -$82.4m*  Deaths averted 1,535  Calibrated to 1,000 cases:  Costs -$423,730*  Deaths averted 7.9 | NR | NR | Results sensitive to Rt; admission, ICU and mortality rates; C+I effect on admissions. However, conclusions remain the same.  PSA: C+I dominant in more than 90% of simulations. | [With C+I] hospitals can achieve important cost savings … while patients can experience a more favourable disease course [including reduction in death]. | Epidemiological model based on estimated parameters, including Rt. Limited clinical evidence about C+I (1 RCT). True price of C+I in Italy is not known, therefore this analysis uses the US price. Dominant COVID-19 variants at the time of publication (alpha and delta) are not the variant that C+I is likely to be active against (omicron; prevalence 4.76%). |
| Savinkina 2022 (20) | Base-case (high) effect scenario, calibrated to 1,000 patients:  No N+R: $221K, 0.77 deaths.  N+R for unvacc high risk: $182K, 0.51 deaths.  N+R for all high risk: 0.29 deaths, $273K.  N+R for all high risk and unvacc low risk: 0.22 deaths, $348K.  N+R for all: 0.18 deaths, $566K. | Base-case (high) effect scenario:  No N+R: baseline.  N+R for unvacc high risk: dominant.  N+R for all high risk: $397K per DA.  N+R for all high risk and unvacc low risk: $1.0m per DA.  N+R for all: $5.0m per DA. | $10,000 to $5m per DA | ICERs, low-effect scenario:  No N+R: baseline.  N+R for unvacc high risk: $319K per DA.  N+R for all high risk: $2.6m per DA.  N+R for all high risk and unvacc low risk: $5.3m per DA.  N+R for all: $22.1m per DA.  Cost results reported for various OWSA values (but ICERs NR). | For almost every scenario … prescribing N+R to unvaccinated patients at high risk of severe COVID-19 was cost saving. This group should almost always be treated if treatment is available. | Analysis does not consider drug supply, budgetary constraints, non-adherence, contraindications to N+R, other active treatments, differential costs in different vacc and risk groups, or transmission dynamics. |
| Shah 2023 (21) | Not reported (incremental only) | Advanced CC vs none:  $186 per DALY averted  Essential CC vs none:  $37 per DALY averted  Advanced CC vs district:  $144 per DALY averted  Essential CC vs district:  $14 per DALY averted | $101 per DALY averted (conservative threshold for Tanzania) | Probability of essential and emergency care being cost effective is 96% and 99% compared to no care and district level care at Tanzanian threshold.  In deterministic analyses, results were most sensitive to effectiveness of essential and emergency care in preventing severe cases becoming critical, unit costs of advanced care | Essential and emergency critical care is likely to be highly cost effective in low-resource settings. | Anlaysis relies on low quality sources for parameters due to scarcity of data, does not include needs of moderate patients, and did not reflect availability of regional and referral hospitals.  Markov model cannot capture pace of change of treatment even within 24 hour cycle.  Triangular distributions used may be less appropriate but reflect uncertain nature of data. |
| Yeung 2022 (10) | Costs and QALYs  Healthcare perspective  Molnupiravir (Mol): $298.5K, 15.938.  N+R: $298.5K, 15.964.  Fluvoxamine (Flu): $297.8K, 15.939.  SoC: $297.7K, 15.925.  Modified societal perspective  Mol: $301.4K, 15.952.  N+R: $302.3K, 16.006.  Flu: $300.8K, 15.954.  SoC: $300.2K, 15.925. | ICERs vs. SoC  Healthcare perspective  Mol: $61K.  N+R: $21K.  Flu: $8K.  Modified societal perspective (approx.)  Mol: $43K.  N+R: $26K.  Flu: $20K. | $50K-150K per QALY gained. | PSA (healthcare perspective), probability ICER < $50K, $100K, $150K:  Mol: 31%, 69%, 84%.  N+R: 97%, 100%, 100%.  Flu: 100%, 100%, 100%.  Key scenarios, (ICERs vs. SoC):  Unvaccinated population:  Mol: $48K.  N+R: $15K.  Flu: $4K.  Lower hospitalisation risk (e.g., Omicron variant):  Mol: $74K.  N+R: $34K.  Flu: $21K. | At their current prices, each intervention is estimated to meet standard cost-effectiveness levels in the US healthcare system, even under a scenario with a lower hospitalisation risk that may reflect the Omicron wave. | Analysis underpinned by immature evidence base and heterogenous trial designs, including non-US settings and different prevalent COVID-19 variants.  Modified societal perspective has limited scope. |
| Abbreviations: Bar, baricitinib; B+R, baricitinib and remdesivir; CC, critical care; CUA, cost—utility analysis; C+I, casirivimab + imdevimab; DALY, disability-adjusted life-year; DA, DALY averted; DCUA, distributional cost—utility analysis; Dex, dexamethasone; DRG, diagnostic-related group; Flu, fluvoxamine; HR, hazard ratio; Hyd, hydroxychloroquine; ICER, incremental cost-effectiveness ratio; ICU, intensive care unit; IF, interferon beta-1a; L+R, lopinavir + ritonivir; Mol, molnupiravir; N+R, nirmatrelvir + ritonavir; NR, not reported; QALY, quality-adjusted life-year; RCT, randomised controlled trial; Rt, disease reproduction rate; R+D, remdesivir and dexamethasone; SoC, standard of care; Sot, sotrovimab; SWQ, south-west quadrant (of the cost-effectiveness plane, i.e., lower cost and lower effectiveness); Toc, tocilizumab. | | | | | | |
| Notes: (1) This study (19) reports the cost results for "static" and "decreasing" infection rate scenarios the other way around, such that the cost in the “static” scenario is lower than the cost under decreasing infection rates. This appears to be an error, therefore we have swapped the cost results. | | | | | | |
| * Cost conversions to USD listed below. The OECD exchange rate for the reported price year is used (29). Where no price-year is explicitly reported, we have assumed the relevant exchange rate is the year prior to the year of publication.   - Alamer 2023 (14): 1 USD = 3.750 SAR (2020) - Lau 2022 (17): 1 USD = 1.341 CAD (2020) - Metry 2022 (9), Rafia 2022 (8): 1 USD = 0.727 GBP (2021) - Ruggeri 2022 (18): 1 USD = 0.845 EUR (2021) - Ruggeri 2023 (22): 1 USD = 0.950 EUR (2022) | | | | | | |
