# Supplementary material for: Diagnostics and treatments of COVID-19: two-year update to a living systematic review of economic evaluations
Source: Front Pharmacol. 2023 Nov 16;14:1291164. doi: 10.3389/fphar.2023.1291164 (PMC10687367; doi:10.3389/fphar.2023.1291164)
Supplement: Supplementary file 2 [file DataSheet1.DOCX]

Supplementary materials

# Search history

| MEDLINE | 2604 |
| --- | --- |
| Medline in Process (Ovid) | 78 |
| Medline epubs (Ovid) | 883 |
| EMBASE | 7916 |
| Cochrane Database of Systematic Reviews (CDSR) | 0 |
| Econlit | 485 |
| INAHTA | 142 |
| Total records | 12108 |
| Total after removing duplicates | 8287 |

# Search strategies

| **Database: MEDLINE** |
| --- |
| Database: Ovid MEDLINE(R) <1946 to March 11, 2021>  Search Strategy:  --------------------------------------------------------------------------------  1 exp coronavirus/ (62203)  2 exp Coronavirus Infections/ (74245)  3 ((corona* or corono*) adj1 (virus* or viral* or virinae*)).ti,ab,kw,kf. (1823)  4 (coronavirus* or coronovirus* or coronavirinae* or CoV).ti,ab,kw,kf. (46216)  5 ("2019-nCoV*" or 2019nCoV* or "19-nCoV*" or 19nCoV* or nCoV2019* or "nCoV-2019*" or nCoV19* or "nCoV-19*" or "COVID-19*" or COVID19* or "COVID-2019*" or COVID2019* or "HCoV-19*" or HCoV19* or "HCoV-2019*" or HCoV2019* or "2019 novel*" or Ncov* or "n-cov" or "SARS-CoV-2*" or "SARSCoV-2*" or "SARSCoV2*" or "SARS-CoV2*" or SARSCov19* or "SARS-Cov19*" or "SARSCov-19*" or "SARS-Cov-19*" or SARSCov2019* or "SARS-Cov2019*" or "SARSCov-2019*" or "SARS-Cov-2019*" or SARS2* or "SARS-2*" or SARScoronavirus2* or "SARS-coronavirus-2*" or "SARScoronavirus 2*" or "SARS coronavirus2*" or SARScoronovirus2* or "SARS-coronovirus-2*" or "SARScoronovirus 2*" or "SARS coronovirus2*" or covid).ti,ab,kw,kf. (61288)  6 (respiratory* adj2 (symptom* or disease* or illness* or condition*) adj5 (Wuhan* or Hubei* or China* or Chinese* or Huanan*)).ti,ab,kw,kf. (262)  7 (("seafood market*" or "food market*") adj10 (Wuhan* or Hubei* or China* or Chinese* or Huanan*)).ti,ab,kw,kf. (75)  8 (pneumonia* adj3 (Wuhan* or Hubei* or China* or Chinese* or Huanan*)).ti,ab,kw,kf. (441)  9 ((outbreak* or wildlife* or pandemic* or epidemic*) adj1 (Wuhan* or Hubei* or China* or Chinese* or Huanan*)).ti,ab,kw,kf. (265)  10 "severe acute respiratory syndrome*".ti,ab,kw,kf. (12330)  11 or/1-10 (85706)  12 limit 11 to yr="2020 -Current" (66433)  13 (sensitiv: or predictive value:).mp. or accurac:.tw. (1940851)  14 Anti-Bacterial Agents/ (345573)  15 (anti-bacter* or antibacter* or "anti bacter*" or anti-mycobacter* or antimycobacter* or "anti mycobacter*" or antibod* or bacteriocid*).ti,ab. (878740)  16 Remdesivir*.ti,ab. (551)  17 (Dexamethasone or 7s5i7g3jql or decaject or decaject or decameth or decaspray or dexasone or dexpak or hexadecadrol or hexadrol or maxidex or methylfluorprednisolone or millicorten or oradexon).ti,ab. (52783)  18 Dexamethasone/ (52118)  19 Adrenal Cortex Hormones/ (65638)  20 (corticosteroid* or corticoid* or (cortex adj4 hormon*)).ti,ab. (99955)  21 bamlanivimab.ti,ab. (4)  22 casirivimab.ti,ab. (3)  23 imdevimab.ti,ab. (3)  24 prednisone.ti,ab. (25184)  25 methylprednisolone.ti,ab. (14492)  26 hydrocortisone.ti,ab. (15532)  27 tocilizumab.ti,ab. (2758)  28 siltuximab.ti,ab. (110)  29 Interleukin-6/ (65869)  30 (Interleukin-6 or "Interleukin 6" or il-6 or il6 or "interferon beta 2" or "interferon beta-2" or "ifn-beta 2" or "hybridoma growth factor" or "hepatocyte stimulating factor" or "b cell differentiat*" or "b cell stimulat*" or "hepatocyte stimulat*" or "hybridoma growth factor*" or "plasmacytoma growth factor").ti,ab. (130560)  31 sarilumab.ti,ab. (96)  32 etesevimab.ti,ab. (1)  33 ((convales* or donate* or donation* or high-titre* or "high titre*") adj4 plasm*).ti,ab. (1249)  34 Baricitinib.ti,ab. (235)  35 Ivermectin.ti,ab. (5452)  36 Anakinra.ti,ab. (1502)  37 (Regdanvimab or CT-P59).ti,ab. (2)  38 Immunoglobulins, Intravenous/ (13746)  39 ((intraven* or IV) adj4 immunoglob*).ti,ab. (12984)  40 (alphaglobin* or endobulin* or flebogamma* or gamimmune* or gamimmune* or gamimune* or gammagard* or gammonativ* or gamunex* or "globulin n" or globulin-n or ivig or intraglobin* or iveegam* or privigen* or sandoglobulin* or venimmune* or venoglobulin*).ti,ab. (6715)  41 (Mesenchym* adj4 stem cell adj4 transplant*).ti,ab. (734)  42 Mesenchymal Stem Cell Transplantation/ (12448)  43 Remestemcel-L.ti,ab. (7)  44 Vitamin D/ (36257)  45 Ascorbic Acid/ (42630)  46 ("Vitamin C" or "Vitamin D").ti,ab. (73834)  47 (Colchicine or "54192-66-4" or "64-86-8" or "75520-89-7" or sml2y3j35t).ti,ab. (14467)  48 Colchicine/ (14230)  49 Azithromycin/ (5770)  50 (Azithromycin or "117772-70-0" or "121470-24-4" or 5fd1131i7s or "83905-01-5" or azadose* or azitrocin* or azythromycin* or "cp 62993" or cp-62993 or cp62993 or goxal* or jte4mnn1md* or sumamed* or toraseptol* or ultreon* or vinzam* or zentavion* or zithromax* or zitromax*).ti,ab. (7890)  51 Doxycycline/ (9941)  52 (doxycycline or "17086-28-1" or 19xts3t51u or "564-25-0").ti,ab. (12315)  53 Lopinavir/ (2046)  54 (Lopinavir* or 2494g1jf75 or "a 157378.0" or a-157378* or a157378* or "abt 378" or abt-378 or abt378 or pydrimidineacetamide*).ti,ab. (2780)  55 Ritonavir/ (4605)  56 (ritonavir* or "abt 538" or abt-538 or abt538 or norvir* or o3j8g9o825).ti,ab. (6198)  57 Hydroxychloroquine/ (4427)  58 Chloroquine/ (14743)  59 (Hydroxychloro* or oxychloro* or chloroquine* or 886u3h6uff or aralen or arechine or arequin or chingamin or chlorochin or khingamin or nivaquine or oe48649k6n or plaquenil or 8q2869cnvh).ti,ab. (21085)  60 Acetaminophen/ (18646)  61 (paracetamol* or "103-90-2" or acetaminophen or 362o9itl9d or apap or acamol or acephen or acetaco or acetamidophenol or acetaminophen or algotropyl or "anacin 3" or anacin-3 or anacin3 or datril or hydroxyacetanilide or panadol or tylenol).ti,ab. (23065)  62 Ibuprofen/ (9075)  63 (ibuprofen or "15687-27-1" or brufen or ibumetin or motrin or nuprin or rufen or salprofen or "trauma dolgit gel" or "trauma-dolgit gel" or wk2xyi10qm).ti,ab. (12420)  64 Anti-Inflammatory Agents, Non-Steroidal/ (68336)  65 Antipyretics/ (2722)  66 (antipyretic* or "anti pyretic*" or antifebril* or "anti febril*").ti,ab. (4903)  67 Analgesics/ (49516)  68 (analgesic* or antinociceptiv* or anodyne*).ti,ab. (81521)  69 or/13-68 (3600419)  70 12 and 69 (10082)  71 Economics/ (27299)  72 exp "Costs and Cost Analysis"/ (243133)  73 Economics, Dental/ (1915)  74 exp Economics, Hospital/ (24993)  75 exp Economics, Medical/ (14245)  76 Economics, Nursing/ (4002)  77 Economics, Pharmaceutical/ (2978)  78 Budgets/ (11401)  79 exp Models, Economic/ (15482)  80 Markov Chains/ (14827)  81 Monte Carlo Method/ (29156)  82 Decision Trees/ (11437)  83 (simulat* adj4 model*).tw. (48398)  84 econom$.tw. (252494)  85 cba.tw. (9910)  86 cea.tw. (21064)  87 cua.tw. (1028)  88 markov$.tw. (18840)  89 (monte adj carlo).tw. (31023)  90 (decision adj3 (tree$ or analys$)).tw. (14444)  91 (cost or costs or costing$ or costly or costed).tw. (481596)  92 (price$ or pricing$).tw. (34896)  93 budget$.tw. (24498)  94 expenditure$.tw. (51179)  95 (value adj3 (money or monetary)).tw. (2199)  96 (pharmacoeconomic$ or (pharmaco adj economic$)).tw. (3550)  97 or/71-96 (1004340)  98 Cost-Benefit Analysis/ (83666)  99 (cost* and ((qualit* adj2 adjust* adj2 life*) or qaly*)).tw. (11542)  100 ((incremental* adj2 cost*) or ICER).tw. (11922)  101 (cost adj2 utilit*).tw. (4600)  102 (cost* and ((net adj benefit*) or (net adj monetary adj benefit*) or (net adj health adj benefit*))).tw. (1483)  103 ((cost adj2 (effect* or utilit*)) and (quality adj of adj life)).tw. (15930)  104 (cost and (effect* or utilit*)).ti. (27713)  105 or/98-104 (94340)  106 "Quality of Life"/ (205545)  107 quality of life.tw. (242905)  108 "Value of Life"/ (5736)  109 Quality-Adjusted Life Years/ (12984)  110 quality adjusted life.tw. (11562)  111 (qaly$ or qald$ or qale$ or qtime$).tw. (9467)  112 disability adjusted life.tw. (2986)  113 daly$.tw. (2697)  114 Health Status Indicators/ (23717)  115 (sf36 or sf 36 or short form 36 or shortform 36 or sf thirtysix or sf thirty six or shortform thirtysix or shortform thirty six or short form thirtysix or short form thirty six).tw. (23271)  116 (sf6 or sf 6 or short form 6 or shortform 6 or sf six or sfsix or shortform six or short form six).tw. (1370)  117 (sf12 or sf 12 or short form 12 or shortform 12 or sf twelve or sftwelve or shortform twelve or short form twelve).tw. (5205)  118 (sf16 or sf 16 or short form 16 or shortform 16 or sf sixteen or sfsixteen or shortform sixteen or short form sixteen).tw. (30)  119 (sf20 or sf 20 or short form 20 or shortform 20 or sf twenty or sftwenty or shortform twenty or short form twenty).tw. (390)  120 (euroqol or euro qol or eq5d or eq 5d).tw. (9817)  121 (qol or hql or hqol or hrqol).tw. (46840)  122 (hye or hyes).tw. (63)  123 health$ year$ equivalent$.tw. (38)  124 utilit$.tw. (179495)  125 (hui or hui1 or hui2 or hui3).tw. (1362)  126 disutili$.tw. (411)  127 rosser.tw. (95)  128 quality of wellbeing.tw. (20)  129 quality of well-being.tw. (393)  130 qwb.tw. (193)  131 willingness to pay.tw. (4880)  132 standard gamble$.tw. (796)  133 time trade off.tw. (1067)  134 time tradeoff.tw. (242)  135 tto.tw. (939)  136 or/106-135 (516804)  137 97 or 105 or 136 (1448798)  138 70 and 137 (759)  139 animals/ not humans/ (4765255)  140 138 not 139 (724)  141 limit 140 to english language (713) |

| **Database: EMBASE** |
| --- |
| Database: Embase <1974 to 2021 March 11>  Search Strategy:  --------------------------------------------------------------------------------  1 exp Coronavirinae/ (23292)  2 exp Coronavirus infection/ (24492)  3 ("coronavirus disease 2019" or "severe acute respiratory syndrome coronavirus 2").sh,dj. (97375)  4 ((corona* or corono*) adj1 (virus* or viral* or virinae*)).ti,ab,kw. (2460)  5 (coronavirus* or coronovirus* or coronavirinae* or CoV).ti,ab,kw. (73586)  6 ("2019-nCoV*" or 2019nCoV* or "19-nCoV*" or 19nCoV* or nCoV2019* or "nCoV-2019*" or nCoV19* or "nCoV-19*" or "COVID-19*" or COVID19* or "COVID-2019*" or COVID2019* or "HCoV-19*" or HCoV19* or "HCoV-2019*" or HCoV2019* or "2019 novel*" or Ncov* or "n-cov" or "SARS-CoV-2*" or "SARSCoV-2*" or "SARSCoV2*" or "SARS-CoV2*" or SARSCov19* or "SARS-Cov19*" or "SARSCov-19*" or "SARS-Cov-19*" or SARSCov2019* or "SARS-Cov2019*" or "SARSCov-2019*" or "SARS-Cov-2019*" or SARS2* or "SARS-2*" or SARScoronavirus2* or "SARS-coronavirus-2*" or "SARScoronavirus 2*" or "SARS coronavirus2*" or SARScoronovirus2* or "SARS-coronovirus-2*" or "SARScoronovirus 2*" or "SARS coronovirus2*" or covid).ti,ab,kw. (105663)  7 (respiratory* adj2 (symptom* or disease* or illness* or condition*) adj5 (Wuhan* or Hubei* or China* or Chinese* or Huanan*)).ti,ab,kw. (398)  8 (("seafood market*" or "food market*") adj10 (Wuhan* or Hubei* or China* or Chinese* or Huanan*)).ti,ab,kw. (108)  9 (pneumonia* adj3 (Wuhan* or Hubei* or China* or Chinese* or Huanan*)).ti,ab,kw. (641)  10 ((outbreak* or wildlife* or pandemic* or epidemic*) adj1 (Wuhan* or Hubei* or China* or Chinese* or Huanan*)).ti,ab,kw. (174)  11 "severe acute respiratory syndrome*".ti,ab,kw. (18023)  12 or/1-11 (142022)  13 limit 12 to yr="2020 -Current" (117010)  14 limit 13 to medline (27451)  15 13 not 14 (89559)  16 (sensitiv: or predictive value:).mp. or accurac:.tw. (2705427)  17 antiinfective agent/ (182635)  18 (anti-bacter* or antibacter* or "anti bacter*" or anti-mycobacter* or antimycobacter* or "anti mycobacter*" or antibod* or bacteriocid*).ti,ab. (1224897)  19 Remdesivir/ (2851)  20 Remdesivir*.ti,ab. (1149)  21 (Dexamethasone or 7s5i7g3jql or decaject or decaject or decameth or decaspray or dexasone or dexpak or hexadecadrol or hexadrol or maxidex or methylfluorprednisolone or millicorten or oradexon).ti,ab. (80234)  22 Dexamethasone/ (155233)  23 corticosteroid/ (241400)  24 (corticosteroid* or corticoid* or (cortex adj4 hormon*)).ti,ab. (164471)  25 monoclonal antibody/ (210770)  26 bamlanivimab.ti,ab. (13)  27 casirivimab.ti,ab. (6)  28 imdevimab.ti,ab. (7)  29 prednisone/ (174954)  30 prednisone.ti,ab. (50030)  31 methylprednisolone/ (99165)  32 methylprednisolone.ti,ab. (26893)  33 hydrocortisone/ (127945)  34 hydrocortisone.ti,ab. (19353)  35 tocilizumab/ (15157)  36 tocilizumab.ti,ab. (8994)  37 siltuximab/ (833)  38 siltuximab.ti,ab. (273)  39 interleukin 6/ (262851)  40 (Interleukin-6 or "Interleukin 6" or il-6 or il6 or "interferon beta 2" or "interferon beta-2" or "ifn-beta 2" or "hybridoma growth factor" or "hepatocyte stimulating factor" or "b cell differentiat*" or "b cell stimulat*" or "hepatocyte stimulat*" or "hybridoma growth factor*" or "plasmacytoma growth factor").ti,ab. (219022)  41 sarilumab/ (957)  42 sarilumab.ti,ab. (441)  43 etesevimab/ (6)  44 etesevimab.ti,ab. (1)  45 plasma transfusion/ (5233)  46 ((convales* or donate* or donation* or high-titre* or "high titre*") adj4 plasm*).ti,ab. (2281)  47 Baricitinib/ (1717)  48 Baricitinib.ti,ab. (969)  49 Ivermectin/ (12950)  50 Ivermectin.ti,ab. (7616)  51 Anakinra/ (3271)  52 Anakinra.ti,ab. (3899)  53 Regdanvimab/ (5)  54 (Regdanvimab or CT-P59).ti,ab. (3)  55 immunoglobulin/ (119861)  56 ((intraven* or IV) adj4 immunoglob*).ti,ab. (24545)  57 (alphaglobin* or endobulin* or flebogamma* or gamimmune* or gamimmune* or gamimune* or gammagard* or gammonativ* or gamunex* or "globulin n" or globulin-n or ivig or intraglobin* or iveegam* or privigen* or sandoglobulin* or venimmune* or venoglobulin*).ti,ab. (18788)  58 (Mesenchym* adj4 stem cell adj4 transplant*).ti,ab. (1706)  59 Mesenchymal Stem Cell Transplantation/ (12423)  60 Remestemcel-L.ti,ab. (20)  61 Vitamin D/ (79519)  62 Ascorbic Acid/ (92473)  63 ("Vitamin C" or "Vitamin D").ti,ab. (122066)  64 (Colchicine or "54192-66-4" or "64-86-8" or "75520-89-7" or sml2y3j35t).ti,ab. (19196)  65 Colchicine/ (31496)  66 Azithromycin/ (39911)  67 (Azithromycin or "117772-70-0" or "121470-24-4" or 5fd1131i7s or "83905-01-5" or azadose* or azitrocin* or azythromycin* or "cp 62993" or cp-62993 or cp62993 or goxal* or jte4mnn1md* or sumamed* or toraseptol* or ultreon* or vinzam* or zentavion* or zithromax* or zitromax*).ti,ab. (14724)  68 Doxycycline/ (55030)  69 (doxycycline or "17086-28-1" or 19xts3t51u or "564-25-0").ti,ab. (20573)  70 Lopinavir/ (7736)  71 (Lopinavir* or 2494g1jf75 or "a 157378.0" or a-157378* or a157378* or "abt 378" or abt-378 or abt378 or pydrimidineacetamide*).ti,ab. (4527)  72 Ritonavir/ (20461)  73 (ritonavir* or "abt 538" or abt-538 or abt538 or norvir* or o3j8g9o825).ti,ab. (10402)  74 Hydroxychloroquine/ (30991)  75 Chloroquine/ (38523)  76 (Hydroxychloro* or oxychloro* or chloroquine* or 886u3h6uff or aralen or arechine or arequin or chingamin or chlorochin or khingamin or nivaquine or oe48649k6n or plaquenil or 8q2869cnvh).ti,ab. (31965)  77 paracetamol/ (94688)  78 (paracetamol* or "103-90-2" or acetaminophen or 362o9itl9d or apap or acamol or acephen or acetaco or acetamidophenol or acetaminophen or algotropyl or "anacin 3" or anacin-3 or anacin3 or datril or hydroxyacetanilide or panadol or tylenol).ti,ab. (42601)  79 Ibuprofen/ (51963)  80 (ibuprofen or "15687-27-1" or brufen or ibumetin or motrin or nuprin or rufen or salprofen or "trauma dolgit gel" or "trauma-dolgit gel" or wk2xyi10qm).ti,ab. (20133)  81 nonsteroid antiinflammatory agent/ (126421)  82 antipyretic agent/ (6362)  83 (antipyretic* or "anti pyretic*" or antifebril* or "anti febril*").ti,ab. (8470)  84 analgesic agent/ (89977)  85 (analgesic* or antinociceptiv* or anodyne*).ti,ab. (130736)  86 or/16-85 (5544406)  87 exp Health Economics/ (876842)  88 exp "Health Care Cost"/ (300516)  89 exp Pharmacoeconomics/ (207604)  90 Monte Carlo Method/ (42496)  91 Decision Tree/ (14429)  92 econom$.tw. (396460)  93 cba.tw. (13088)  94 cea.tw. (36208)  95 cua.tw. (1592)  96 markov$.tw. (32524)  97 (monte adj carlo).tw. (51267)  98 (decision adj3 (tree$ or analys$)).tw. (25988)  99 (cost or costs or costing$ or costly or costed).tw. (821532)  100 (price$ or pricing$).tw. (60881)  101 budget$.tw. (40633)  102 expenditure$.tw. (78414)  103 (value adj3 (money or monetary)).tw. (3643)  104 (pharmacoeconomic$ or (pharmaco adj economic$)).tw. (8879)  105 or/87-104 (1858937)  106 "Quality of Life"/ (500289)  107 Quality Adjusted Life Year/ (28486)  108 Quality of Life Index/ (2862)  109 Short Form 36/ (31350)  110 Health Status/ (132567)  111 quality of life.tw. (471985)  112 quality adjusted life.tw. (21198)  113 (qaly$ or qald$ or qale$ or qtime$).tw. (21575)  114 disability adjusted life.tw. (4520)  115 daly$.tw. (4399)  116 (sf36 or sf 36 or short form 36 or shortform 36 or sf thirtysix or sf thirty six or shortform thirtysix or shortform thirty six or short form thirtysix or short form thirty six).tw. (43576)  117 (sf6 or sf 6 or short form 6 or shortform 6 or sf six or sfsix or shortform six or short form six).tw. (2544)  118 (sf12 or sf 12 or short form 12 or shortform 12 or sf twelve or sftwelve or shortform twelve or short form twelve).tw. (10058)  119 (sf16 or sf 16 or short form 16 or shortform 16 or sf sixteen or sfsixteen or shortform sixteen or short form sixteen).tw. (64)  120 (sf20 or sf 20 or short form 20 or shortform 20 or sf twenty or sftwenty or shortform twenty or short form twenty).tw. (464)  121 (euroqol or euro qol or eq5d or eq 5d).tw. (22738)  122 (qol or hql or hqol or hrqol).tw. (104291)  123 (hye or hyes).tw. (140)  124 health$ year$ equivalent$.tw. (41)  125 utilit$.tw. (308858)  126 (hui or hui1 or hui2 or hui3).tw. (2462)  127 disutili$.tw. (987)  128 rosser.tw. (128)  129 quality of wellbeing.tw. (49)  130 quality of well-being.tw. (506)  131 qwb.tw. (255)  132 willingness to pay.tw. (9638)  133 standard gamble$.tw. (1124)  134 time trade off.tw. (1778)  135 time tradeoff.tw. (300)  136 tto.tw. (1784)  137 or/106-136 (1054006)  138 cost utility analysis/ (10223)  139 (cost* and ((qualit* adj2 adjust* adj2 life*) or qaly*)).tw. (24123)  140 ((incremental* adj2 cost*) or ICER).tw. (24746)  141 (cost adj2 utilit*).tw. (8945)  142 (cost* and ((net adj benefit*) or (net adj monetary adj benefit*) or (net adj health adj benefit*))).tw. (2479)  143 ((cost adj2 (effect* or utilit*)) and (quality adj of adj life)).tw. (29549)  144 (cost and (effect* or utilit*)).ti. (48425)  145 or/138-144 (76332)  146 105 or 137 or 145 (2747221)  147 15 and 86 and 146 (1884)  148 nonhuman/ not human/ (4777544)  149 147 not 148 (1779)  150 limit 149 to english language (1751) |

| **Database: CDSR** |
| --- |
| #1 MeSH descriptor: [Coronavirus] explode all trees 217  #2 MeSH descriptor: [Coronavirus Infections] explode all trees 728  #3 ((corona* or corono*) near/1 (virus* or viral* or virinae*)):ti,ab,kw 192  #4 (coronavirus* or coronovirus* or coronavirinae* or CoV):ti,ab,kw 2715  #5 ("2019-nCoV*" or 2019nCoV* or "19-nCoV*" or 19nCoV* or nCoV2019* or "nCoV-2019*" or nCoV19* or "nCoV-19*" or "COVID-19*" or COVID19* or "COVID-2019*" or COVID2019* or "HCoV-19*" or HCoV19* or "HCoV-2019*" or HCoV2019* or "2019 novel*" or Ncov* or "n-cov" or "SARS-CoV-2*" or "SARSCoV-2*" or "SARSCoV2*" or "SARS-CoV2*" or SARSCov19* or "SARS-Cov19*" or "SARSCov-19*" or "SARS-Cov-19*" or SARSCov2019* or "SARS-Cov2019*" or "SARSCov-2019*" or "SARS-Cov-2019*" or SARS2* or "SARS-2*" or SARScoronavirus2* or "SARS-coronavirus-2*" or "SARScoronavirus 2*" or "SARS coronavirus2*" or SARScoronovirus2* or "SARS-coronovirus-2*" or "SARScoronovirus 2*" or "SARS coronovirus2*" or covid):ti,ab,kw 4369  #6 (respiratory* adj2 (symptom* or disease* or illness* or condition*) adj5 (Wuhan* or Hubei* or China* or Chinese* or Huanan*)):ti,ab,kw 0  #7 (("seafood market*" or "food market*") adj10 (Wuhan* or Hubei* or China* or Chinese* or Huanan*)):ti,ab,kw 0  #8 (pneumonia* adj3 (Wuhan* or Hubei* or China* or Chinese* or Huanan*)):ti,ab,kw 0  #9 ((outbreak* or wildlife* or pandemic* or epidemic*) adj1 (Wuhan* or Hubei* or China* or Chinese* or Huanan*)):ti,ab,kw 0  #10 "severe acute respiratory syndrome*":ti,ab,kw 782  #11 {OR #1-#10} with Cochrane Library publication date Between Jan 2020 and Mar 2021, in Cochrane Reviews, Cochrane Protocols 38  #12 (sensitiv* or predictive value* or accurac*):ti,ab,kw 99994  #13 MeSH descriptor: [Anti-Bacterial Agents] this term only 11081  #14 (anti-bacter* or antibacter* or "anti bacter*" or anti-mycobacter* or antimycobacter* or "anti mycobacter*" or antibod* or bacteriocid*):ti,ab 37142  #15 Remdesivir*:ti,ab 126  #16 (Dexamethasone or 7s5i7g3jql or decaject or decaject or decameth or decaspray or dexasone or dexpak or hexadecadrol or hexadrol or maxidex or methylfluorprednisolone or millicorten or oradexon):ti,ab 10766  #17 MeSH descriptor: [Dexamethasone] this term only 4623  #18 MeSH descriptor: [Adrenal Cortex Hormones] this term only 2345  #19 (corticosteroid* or corticoid* or (cortex adj4 hormon*)):ti,ab 21428  #20 bamlanivimab:ti,ab 1  #21 casirivimab:ti,ab 0  #22 imdevimab:ti,ab 0  #23 prednisone:ti,ab 7679  #24 methylprednisolone:ti,ab 3665  #25 hydrocortisone:ti,ab 2415  #26 tocilizumab:ti,ab 1252  #27 siltuximab:ti,ab 64  #28 MeSH descriptor: [Interleukin-6] this term only 3056  #29 (Interleukin-6 or "Interleukin 6" or il-6 or il6 or "interferon beta 2" or "interferon beta-2" or "ifn-beta 2" or "hybridoma growth factor" or "hepatocyte stimulating factor" or "b cell differentiat*" or "b cell stimulat*" or "hepatocyte stimulat*" or "hybridoma growth factor*" or "plasmacytoma growth factor"):ti,ab 15116  #30 sarilumab:ti,ab 261  #31 etesevimab:ti,ab 0  #32 ((convales* or donate* or donation* or high-titre* or "high titre*") adj4 plasm*):ti,ab 0  #33 Baricitinib:ti,ab 413  #34 Ivermectin:ti,ab 708  #35 Anakinra:ti,ab 371  #36 (Regdanvimab or CT-P59):ti,ab 4  #37 MeSH descriptor: [Immunoglobulins, Intravenous] this term only 862  #38 ((intraven* or IV) adj4 immunoglob*):ti,ab 0  #39 (alphaglobin* or endobulin* or flebogamma* or gamimmune* or gamimmune* or gamimune* or gammagard* or gammonativ* or gamunex* or "globulin n" or globulin-n or ivig or intraglobin* or iveegam* or privigen* or sandoglobulin* or venimmune* or venoglobulin*):ti,ab 1499  #40 (Mesenchym* adj4 stem cell adj4 transplant*):ti,ab 0  #41 MeSH descriptor: [Mesenchymal Stem Cell Transplantation] this term only 206  #42 Remestemcel-L:ti,ab 6  #43 MeSH descriptor: [Vitamin D] this term only 3597  #44 MeSH descriptor: [Ascorbic Acid] this term only 2247  #45 ("Vitamin C" or "Vitamin D"):ti,ab 14826  #46 (Colchicine or "54192-66-4" or "64-86-8" or "75520-89-7" or sml2y3j35t):ti,ab 839  #47 MeSH descriptor: [Colchicine] this term only 355  #48 MeSH descriptor: [Azithromycin] this term only 1019  #49 (Azithromycin or "117772-70-0" or "121470-24-4" or 5fd1131i7s or "83905-01-5" or azadose* or azitrocin* or azythromycin* or "cp 62993" or cp-62993 or cp62993 or goxal* or jte4mnn1md* or sumamed* or toraseptol* or ultreon* or vinzam* or zentavion* or zithromax* or zitromax*):ti,ab 2711  #50 MeSH descriptor: [Doxycycline] this term only 1077  #51 (doxycycline or "17086-28-1" or 19xts3t51u or "564-25-0"):ti,ab 1942  #52 MeSH descriptor: [Lopinavir] this term only 562  #53 (Lopinavir* or 2494g1jf75 or "a 157378.0" or a-157378* or a157378* or "abt 378" or abt-378 or abt378 or pydrimidineacetamide*):ti,ab 1134  #54 MeSH descriptor: [Ritonavir] this term only 1289  #55 (ritonavir* or "abt 538" or abt-538 or abt538 or norvir* or o3j8g9o825):ti,ab 2743  #56 MeSH descriptor: [Hydroxychloroquine] this term only 524  #57 MeSH descriptor: [Chloroquine] this term only 718  #58 (Hydroxychloro* or oxychloro* or chloroquine* or 886u3h6uff or aralen or arechine or arequin or chingamin or chlorochin or khingamin or nivaquine or oe48649k6n or plaquenil or 8q2869cnvh):ti,ab 2749  #59 MeSH descriptor: [Acetaminophen] this term only 3297  #60 (paracetamol* or "103-90-2" or acetaminophen or 362o9itl9d or apap or acamol or acephen or acetaco or acetamidophenol or acetaminophen or algotropyl or "anacin 3" or anacin-3 or anacin3 or datril or hydroxyacetanilide or panadol or tylenol):ti,ab 9180  #61 MeSH descriptor: [Ibuprofen] this term only 2013  #62 (ibuprofen or "15687-27-1" or brufen or ibumetin or motrin or nuprin or rufen or salprofen or "trauma dolgit gel" or "trauma-dolgit gel" or wk2xyi10qm):ti,ab 4451  #63 MeSH descriptor: [Anti-Inflammatory Agents, Non-Steroidal] this term only 6578  #64 MeSH descriptor: [Antipyretics] this term only 82  #65 (antipyretic* or "anti pyretic*" or antifebril* or "anti febril*"):ti,ab 890  #66 MeSH descriptor: [Analgesics] this term only 5144  #67 (analgesic* or antinociceptiv* or anodyne*):ti,ab 34869  #68 {OR #12-#67} 263399  #69 #11 AND #68 15 |

| **Database: EconLit** |
| --- |
| 1 ((corona* or corono*) adj1 (virus* or viral* or virinae*)).ti,ab,kw. (13)  2 (coronavirus* or coronovirus* or coronavirinae* or CoV).ti,ab,kw. (527)  3 ("2019-nCoV*" or 2019nCoV* or "19-nCoV*" or 19nCoV* or nCoV2019* or "nCoV-2019*" or nCoV19* or "nCoV-19*" or "COVID-19*" or COVID19* or "COVID-2019*" or COVID2019* or "HCoV-19*" or HCoV19* or "HCoV-2019*" or HCoV2019* or "2019 novel*" or Ncov* or "n-cov" or "SARS-CoV-2*" or "SARSCoV-2*" or "SARSCoV2*" or "SARS-CoV2*" or SARSCov19* or "SARS-Cov19*" or "SARSCov-19*" or "SARS-Cov-19*" or SARSCov2019* or "SARS-Cov2019*" or "SARSCov-2019*" or "SARS-Cov-2019*" or SARS2* or "SARS-2*" or SARScoronavirus2* or "SARS-coronavirus-2*" or "SARScoronavirus 2*" or "SARS coronavirus2*" or SARScoronovirus2* or "SARS-coronovirus-2*" or "SARScoronovirus 2*" or "SARS coronovirus2*" or covid).ti,ab,kw. (2348)  4 (respiratory* adj2 (symptom* or disease* or illness* or condition*) adj5 (Wuhan* or Hubei* or China* or Chinese* or Huanan*)).ti,ab,kw. (0)  5 (("seafood market*" or "food market*") adj10 (Wuhan* or Hubei* or China* or Chinese* or Huanan*)).ti,ab,kw. (31)  6 (pneumonia* adj3 (Wuhan* or Hubei* or China* or Chinese* or Huanan*)).ti,ab,kw. (0)  7 ((outbreak* or wildlife* or pandemic* or epidemic*) adj1 (Wuhan* or Hubei* or China* or Chinese* or Huanan*)).ti,ab,kw. (4)  8 "severe acute respiratory syndrome*".ti,ab,kw. (40)  9 or/1-8 (2578)  10 limit 9 to yr="2020 -Current" (2494)  11 (sensitiv: or predictive value:).mp. or accurac:.tw. (35355)  12 (anti-bacter* or antibacter* or "anti bacter*" or anti-mycobacter* or antimycobacter* or "anti mycobacter*" or antibod* or bacteriocid*).ti,ab. (63)  13 Remdesivir*.ti,ab. (0)  14 (Dexamethasone or 7s5i7g3jql or decaject or decaject or decameth or decaspray or dexasone or dexpak or hexadecadrol or hexadrol or maxidex or methylfluorprednisolone or millicorten or oradexon).ti,ab. (4)  15 (corticosteroid* or corticoid* or (cortex adj4 hormon*)).ti,ab. (17)  16 bamlanivimab.ti,ab. (0)  17 casirivimab.ti,ab. (0)  18 imdevimab.ti,ab. (0)  19 prednisone.ti,ab. (2)  20 methylprednisolone.ti,ab. (0)  21 hydrocortisone.ti,ab. (0)  22 tocilizumab.ti,ab. (0)  23 siltuximab.ti,ab. (0)  24 (Interleukin-6 or "Interleukin 6" or il-6 or il6 or "interferon beta 2" or "interferon beta-2" or "ifn-beta 2" or "hybridoma growth factor" or "hepatocyte stimulating factor" or "b cell differentiat*" or "b cell stimulat*" or "hepatocyte stimulat*" or "hybridoma growth factor*" or "plasmacytoma growth factor").ti,ab. (1)  25 sarilumab.ti,ab. (0)  26 etesevimab.ti,ab. (0)  27 ((convales* or donate* or donation* or high-titre* or "high titre*") adj4 plasm*).ti,ab. (7)  28 Baricitinib.ti,ab. (0)  29 Ivermectin.ti,ab. (1)  30 Anakinra.ti,ab. (0)  31 (Regdanvimab or CT-P59).ti,ab. (0)  32 ((intraven* or IV) adj4 immunoglob*).ti,ab. (2)  33 (alphaglobin* or endobulin* or flebogamma* or gamimmune* or gamimmune* or gamimune* or gammagard* or gammonativ* or gamunex* or "globulin n" or globulin-n or ivig or intraglobin* or iveegam* or privigen* or sandoglobulin* or venimmune* or venoglobulin*).ti,ab. (2)  34 (Mesenchym* adj4 stem cell adj4 transplant*).ti,ab. (0)  35 Remestemcel-L.ti,ab. (0)  36 ("Vitamin C" or "Vitamin D").ti,ab. (57)  37 (Colchicine or "54192-66-4" or "64-86-8" or "75520-89-7" or sml2y3j35t).ti,ab. (0)  38 (Azithromycin or "117772-70-0" or "121470-24-4" or 5fd1131i7s or "83905-01-5" or azadose* or azitrocin* or azythromycin* or "cp 62993" or cp-62993 or cp62993 or goxal* or jte4mnn1md* or sumamed* or toraseptol* or ultreon* or vinzam* or zentavion* or zithromax* or zitromax*).ti,ab. (0)  39 (doxycycline or "17086-28-1" or 19xts3t51u or "564-25-0").ti,ab. (0)  40 (Lopinavir* or 2494g1jf75 or "a 157378.0" or a-157378* or a157378* or "abt 378" or abt-378 or abt378 or pydrimidineacetamide*).ti,ab. (1)  41 (ritonavir* or "abt 538" or abt-538 or abt538 or norvir* or o3j8g9o825).ti,ab. (3)  42 (Hydroxychloro* or oxychloro* or chloroquine* or 886u3h6uff or aralen or arechine or arequin or chingamin or chlorochin or khingamin or nivaquine or oe48649k6n or plaquenil or 8q2869cnvh).ti,ab. (9)  43 (paracetamol* or "103-90-2" or acetaminophen or 362o9itl9d or apap or acamol or acephen or acetaco or acetamidophenol or acetaminophen or algotropyl or "anacin 3" or anacin-3 or anacin3 or datril or hydroxyacetanilide or panadol or tylenol).ti,ab. (11)  44 (ibuprofen or "15687-27-1" or brufen or ibumetin or motrin or nuprin or rufen or salprofen or "trauma dolgit gel" or "trauma-dolgit gel" or wk2xyi10qm).ti,ab. (2)  45 (antipyretic* or "anti pyretic*" or antifebril* or "anti febril*").ti,ab. (1)  46 (analgesic* or antinociceptiv* or anodyne*).ti,ab. (35)  47 or/11-46 (35541)  48 10 and 47 (83) |

| **Database: INAHTA** |
| --- |
| Search query  ((corona* or corono*) AND (virus* or viral* or virinae*))  (coronavirus* or coronovirus* or coronavirinae* or CoV)  ("2019-nCoV*" or 2019nCoV* or "19-nCoV*" or 19nCoV* or nCoV2019* or "nCoV-2019*" or nCoV19* or "nCoV-19*" or "COVID-19*" or COVID19* or "COVID-2019*" or COVID2019* or "HCoV-19*" or HCoV19* or "HCoV-2019*" or HCoV2019* or "2019 novel*" or Ncov* or "n-cov" or "SARS-CoV-2*" or "SARSCoV-2*" or "SARSCoV2*" or "SARS-CoV2*" or SARSCov19* or "SARS-Cov19*" or "SARSCov-19*" or "SARS-Cov-19*" or SARSCov2019* or "SARS-Cov2019*" or "SARSCov-2019*" or "SARS-Cov-2019*" or SARS2* or "SARS-2*" or SARScoronavirus2* or "SARS-coronavirus-2*" or "SARScoronavirus 2*" or "SARS coronavirus2*" or SARScoronovirus2* or "SARS-coronovirus-2*" or "SARScoronovirus 2*" or "SARS coronovirus2*" or covid)  (respiratory* AND (symptom* or disease* or illness* or condition*) AND (Wuhan* or Hubei* or China* or Chinese* or Huanan*))  (("seafood market*" or "food market*") AND (Wuhan* or Hubei* or China* or Chinese* or Huanan*))  (pneumonia* AND (Wuhan* or Hubei* or China* or Chinese* or Huanan*))  ((outbreak* or wildlife* or pandemic* or epidemic*) AND (Wuhan* or Hubei* or China* or Chinese* or Huanan*))  "severe acute respiratory syndrome*"  ("severe acute respiratory syndrome*") OR (((outbreak* or wildlife* or pandemic* or epidemic*) AND (Wuhan* or Hubei* or China* or Chinese* or Huanan*))) OR ((pneumonia* AND (Wuhan* or Hubei* or China* or Chinese* or Huanan*))) OR ((("seafood market*" or "food market*") AND0 (Wuhan* or Hubei* or China* or Chinese* or Huanan*))) OR ((respiratory* AND (symptom* or disease* or illness* or condition*) AND (Wuhan* or Hubei* or China* or Chinese* or Huanan*))) OR (("2019-nCoV*" or 2019nCoV* or "19-nCoV*" or 19nCoV* or nCoV2019* or "nCoV-2019*" or nCoV19* or "nCoV-19*" or "COVID-19*" or COVID19* or "COVID-2019*" or COVID2019* or "HCoV-19*" or HCoV19* or "HCoV-2019*" or HCoV2019* or "2019 novel*" or Ncov* or "n-cov" or "SARS-CoV-2*" or "SARSCoV-2*" or "SARSCoV2*" or "SARS-CoV2*" or SARSCov19* or "SARS-Cov19*" or "SARSCov-19*" or "SARS-Cov-19*" or SARSCov2019* or "SARS-Cov2019*" or "SARSCov-2019*" or "SARS-Cov-2019*" or SARS2* or "SARS-2*" or SARScoronavirus2* or "SARS-coronavirus-2*" or "SARScoronavirus 2*" or "SARS coronavirus2*" or SARScoronovirus2* or "SARS-coronovirus-2*" or "SARScoronovirus 2*" or "SARS coronovirus2*" or covid)) OR ((coronavirus* or coronovirus* or coronavirinae* or CoV)) OR (((corona* or corono*) AND (virus* or viral* or virinae*))) |
